# Inhibition of the programmed death protein 1 immune checkpoint and the development of heart failure in the presence of prior cardiac ischaemia

**DOI:** 10.1093/cvr/cvag085

**Published:** 2026-04-22

**Authors:** Tamás G Gergely, Zsófia D Drobni, Tamás Kovács, Nabil V Sayour, Viktória E Tóth, Márton S Kocsis, Zsófia Onódi, Gábor M Mórotz, Andrea Kovács, Daniel A Zlotoff, Hannah K Gilman, Jingyi Gong, Nóra Fekete, Éva Pállinger, Edit I Buzás, Laura I Yousif, Wouter C Meijers, Béla Merkely, Kerry L Reynolds, Péter Ferdinandy, Tomas G Neilan, Zoltán V Varga

**Affiliations:** Department of Pharmacology and Pharmacotherapy, Semmelweis University, Budapest 1089, Hungary; Center for Pharmacology and Drug Research & Development, Semmelweis University, Budapest 1089, Hungary; HCEMM-SU Cardiometabolic Immunology Research Group, Budapest 1089, Hungary; MTA-SE Momentum Cardio-Oncology and Cardioimmunology Research Group, Budapest 1089, Hungary; Heart and Vascular Center, Semmelweis University, Budapest 1122, Hungary; Cardiovascular Imaging Research Center (CIRC), Department of Radiology and Division of Cardiology, Massachusetts General Hospital, Harvard Medical School, Boston, MA 02114, USA; Department of Pharmacology and Pharmacotherapy, Semmelweis University, Budapest 1089, Hungary; Center for Pharmacology and Drug Research & Development, Semmelweis University, Budapest 1089, Hungary; HCEMM-SU Cardiometabolic Immunology Research Group, Budapest 1089, Hungary; MTA-SE Momentum Cardio-Oncology and Cardioimmunology Research Group, Budapest 1089, Hungary; Department of Pharmacology and Pharmacotherapy, Semmelweis University, Budapest 1089, Hungary; Center for Pharmacology and Drug Research & Development, Semmelweis University, Budapest 1089, Hungary; HCEMM-SU Cardiometabolic Immunology Research Group, Budapest 1089, Hungary; MTA-SE Momentum Cardio-Oncology and Cardioimmunology Research Group, Budapest 1089, Hungary; Department of Pharmacology and Pharmacotherapy, Semmelweis University, Budapest 1089, Hungary; Center for Pharmacology and Drug Research & Development, Semmelweis University, Budapest 1089, Hungary; HCEMM-SU Cardiometabolic Immunology Research Group, Budapest 1089, Hungary; MTA-SE Momentum Cardio-Oncology and Cardioimmunology Research Group, Budapest 1089, Hungary; Department of Pharmacology and Pharmacotherapy, Semmelweis University, Budapest 1089, Hungary; Center for Pharmacology and Drug Research & Development, Semmelweis University, Budapest 1089, Hungary; HCEMM-SU Cardiometabolic Immunology Research Group, Budapest 1089, Hungary; MTA-SE Momentum Cardio-Oncology and Cardioimmunology Research Group, Budapest 1089, Hungary; Department of Pharmacology and Pharmacotherapy, Semmelweis University, Budapest 1089, Hungary; Center for Pharmacology and Drug Research & Development, Semmelweis University, Budapest 1089, Hungary; HCEMM-SU Cardiometabolic Immunology Research Group, Budapest 1089, Hungary; MTA-SE Momentum Cardio-Oncology and Cardioimmunology Research Group, Budapest 1089, Hungary; Department of Pharmacology and Pharmacotherapy, Semmelweis University, Budapest 1089, Hungary; Center for Pharmacology and Drug Research & Development, Semmelweis University, Budapest 1089, Hungary; HCEMM-SU Cardiometabolic Immunology Research Group, Budapest 1089, Hungary; MTA-SE Momentum Cardio-Oncology and Cardioimmunology Research Group, Budapest 1089, Hungary; Department of Pharmacology and Pharmacotherapy, Semmelweis University, Budapest 1089, Hungary; Center for Pharmacology and Drug Research & Development, Semmelweis University, Budapest 1089, Hungary; HCEMM-SU Cardiometabolic Immunology Research Group, Budapest 1089, Hungary; MTA-SE Momentum Cardio-Oncology and Cardioimmunology Research Group, Budapest 1089, Hungary; Cardiovascular Imaging Research Center (CIRC), Department of Radiology and Division of Cardiology, Massachusetts General Hospital, Harvard Medical School, Boston, MA 02114, USA; Cardiovascular Imaging Research Center (CIRC), Department of Radiology and Division of Cardiology, Massachusetts General Hospital, Harvard Medical School, Boston, MA 02114, USA; Cardiovascular Imaging Research Center (CIRC), Department of Radiology and Division of Cardiology, Massachusetts General Hospital, Harvard Medical School, Boston, MA 02114, USA; Department of Genetics, Cell- and Immunobiology, Semmelweis University, Budapest 1089, Hungary; Department of Genetics, Cell- and Immunobiology, Semmelweis University, Budapest 1089, Hungary; Department of Genetics, Cell- and Immunobiology, Semmelweis University, Budapest 1089, Hungary; Department of Cardiology, Thorax Center, Cardiovascular Institute, Erasmus University Medical Center, Rotterdam 3015GD, The Netherlands; Department of Cardiology, Thorax Center, Cardiovascular Institute, Erasmus University Medical Center, Rotterdam 3015GD, The Netherlands; Heart and Vascular Center, Semmelweis University, Budapest 1122, Hungary; Division of Oncology and Hematology, Department of Medicine, Massachusetts General Hospital, Harvard Medical School, Boston, MA 02114, USA; Department of Pharmacology and Pharmacotherapy, Semmelweis University, Budapest 1089, Hungary; Center for Pharmacology and Drug Research & Development, Semmelweis University, Budapest 1089, Hungary; Pharmahungary Group, Szeged 6722, Hungary; Cardiovascular Imaging Research Center (CIRC), Department of Radiology and Division of Cardiology, Massachusetts General Hospital, Harvard Medical School, Boston, MA 02114, USA; Department of Pharmacology and Pharmacotherapy, Semmelweis University, Budapest 1089, Hungary; Center for Pharmacology and Drug Research & Development, Semmelweis University, Budapest 1089, Hungary; HCEMM-SU Cardiometabolic Immunology Research Group, Budapest 1089, Hungary; MTA-SE Momentum Cardio-Oncology and Cardioimmunology Research Group, Budapest 1089, Hungary

**Keywords:** Immune checkpoint inhibitor, Cardiotoxicity, Heart failure, Immune-related adverse event

## Abstract

**Aims:**

Immune checkpoint inhibitors (ICIs) have revolutionized cancer treatment. However, their use often leads to cardiovascular adverse effects, including cardiac dysfunction. Here, we hypothesized that a prior cardiac ischaemic injury could exacerbate cardiac dysfunction due to anti-programmed death protein 1 (PD-1) treatment. Furthermore, we investigated whether abatacept, a T-cell costimulation blocker, could ameliorate the ICI-induced cardiotoxicity in a pre-clinical model.

**Methods and results:**

In a pre-clinical study, mice were treated with isoprenaline or control to induce reversible cardiac ischaemia. After 16 weeks of follow-up, recovery of cardiac function was confirmed via echocardiography, and mice from both groups were randomly treated with isotype control, anti-PD-1, or anti-PD-1 combined with abatacept, for 2 further weeks. Mice with prior ischaemic injury and anti-PD-1 treatment showed cardiac dysfunction with increased infiltration of T cells and macrophages and elevated expression of pro-inflammatory cytokines. Conversely, cardiac dysfunction and inflammation were less pronounced after anti-PD-1 treatment in mice without prior ischaemic injury. Mice with concomitant abatacept treatment exhibited normal cardiac function and alleviated pro-inflammatory response. In a parallel single-centre retrospective clinical cohort study, 1671 cancer patients receiving PD-1 inhibitors were analysed. Cases were defined as patients who developed incident heart failure (HF) after ICI initiation with a primary aim to test whether pre-existing ischaemic heart disease was associated with an increased risk for HF development post-ICI therapy. Sensitivity analyses included propensity score matching and comparison with non-ICI-treated cancer patients. Among ICI-treated patients, 109 (6.5%) developed HF over a median follow-up of 332 days. Multivariable logistic regression of the matched population showed increased odds of incident HF in patients with prior ischaemic cardiac events (odds ratio 2.11, 95% confidence interval 1.05–4.2, *P* = 0.033).

**Conclusion:**

In mice, induction of cardiac inflammation and dysfunction by anti-PD-1 therapy was potentiated by prior transient ischaemic cardiac injury, which was ameliorated by abatacept cotreatment. Cancer patients with pre-existing ischaemic heart disease may be at greater risk for developing ICI-induced new-onset HF. Based on our findings, cardiac surveillance should be considered in patients starting ICI therapy with a prior history of ischaemic heart disease.

## Graphical Abstract

**Graphical Abstract:**
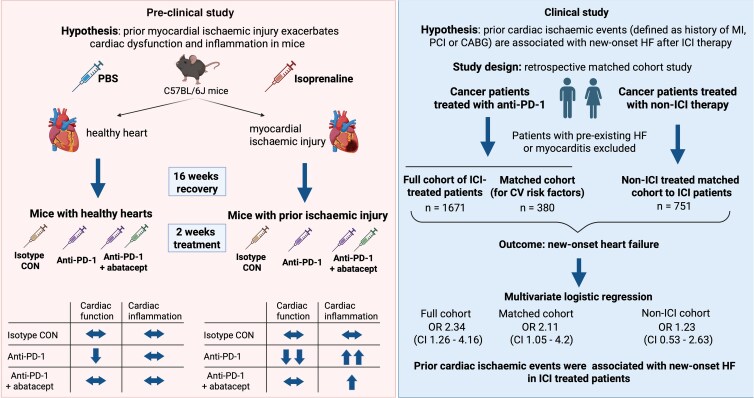

**Time for primary review: 61 days**

## 1. Introduction

Immune checkpoint molecules, such as programmed death protein 1 (PD-1) and its ligand, PD-L1, are physiological regulators of immune cell activation, with a necessary role in maintaining homeostasis and preventing autoimmune responses. Pharmacological targeting of this system with immune checkpoint inhibitors (ICIs) has led to a breakthrough in cancer treatment, and the oncological indications for ICI therapy are rapidly expanding.^1^

However, inhibition of immune checkpoints causes systemic immune activation, leading to immune-related adverse events (irAEs), including various cardiovascular side effects. The spectrum of ICI-induced cardiovascular toxicity is broad and includes myocardial, pericardial,^2^ and vascular disease.^3–5^ Myocardial adverse events after ICI therapy can occur in many forms, including myocarditis,^6^ left ventricular systolic dysfunction without clear myocarditis,^7^ or heart failure.^8^ Of these, ICI-induced fulminant myocarditis is the most well-known and characterized cardiac adverse event, presenting with myocardial immune cell infiltration and is associated with high morbidity and mortality.^6,9^

Recently, cardiac dysfunction or heart failure without overt myocarditis has been reported,^10^ with potential early^11^ or late-onset manifestations.^12^ The risk factors for the development of non-myocarditis heart failure after ICI use are incompletely understood. Previous studies have shown that myocardial ischaemic injury affects the cardiac immune milieu, including immune checkpoint signalling,^13^ and predisposes to ICI-induced myocarditis through myosin-specific tissue-resident memory T-cell activation.^14^ Here, we hypothesized that prior cardiac ischaemic injury may exacerbate the cardiac dysfunction associated with ICIs and may be a risk factor for new-onset heart failure development after ICI treatment.

Abatacept CTLA-4 fusion protein (CTLA-4-Ig), a T-cell costimulation inhibitor, is a potential treatment option in ICI myocarditis, by reducing myocardial immune cell infiltration and inflammation, both in pre-clinical models^15^ and clinical case studies.^16,17^ Moreover, abatacept has been shown to alleviate cardiac dysfunction in pre-clinical heart failure models^18^; however, it is unknown if abatacept has a beneficial effect in non-myocarditis heart failure after ICI therapy.

Thus, in this study, we created a model of ischaemic cardiac injury and tested the role of PD-1 inhibition and concomitant use of abatacept. In a parallel clinical study, we tested whether prior ischaemic cardiac events were a risk factor for ICI-associated heart failure.

## 2. Methods

### 2.1 Experimental animals and ethical statements

For the pre-clinical study, all procedures were approved by the National Scientific Ethical Committee on Animal Experimentation and the Semmelweis University’s Institutional Animal Care and Use Committee (H-1089 Budapest, Hungary) in accordance with NIH guidelines [National Research Council (2011), Guide for the Care and Use of Laboratory Animals: Eighth Edition] and permitted by the government of Food Chain Safety and Animal Health Directorate of the Government Office for Pest County (project identification number: PE/EA/1912-7/2017).

Experimental procedures were carried out on 8–10-week-old, male C57Bl/6J mice (Janvier Labs, Le Genest-Saint-Isle, France), weighing 21–29 g at the beginning of the study. The animals had an acclimatization period of at least 1 week before experiments. Mice were maintained under a 12:12 h light–dark cycle under controlled environmental conditions (20–24°C and 35–75% relative humidity) in individually ventilated cages with shelters, holding two to three animals per cage. Standard rodent chow and tap water were provided ad libitum throughout the entire experiment.

### 2.2 Experimental groups and model of reversible myocardial ischaemic injury

**Figure 1:**
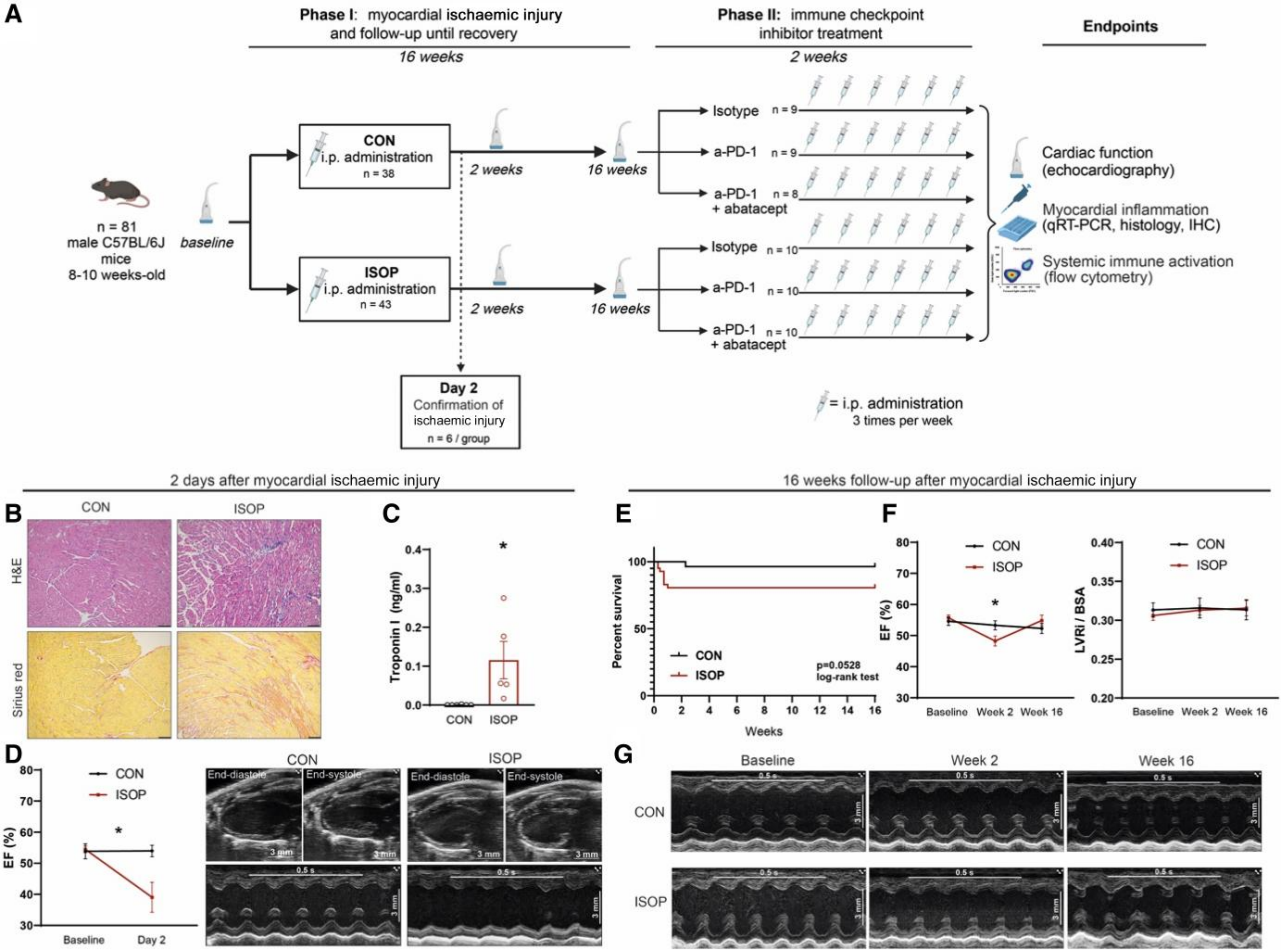
Pre-clinical model of reversible myocardial ischaemic injury. (*A*) Experimental design. Mice were treated with either ISOP or PBS (CON) and followed up for 16 weeks for functional recovery, assessed by echocardiography (Phase I). After this, mice were randomized into six groups, receiving isotype control antibodies, anti-PD-1 antibodies, or anti-PD-1 and abatacept cotreatment (Phase II). (*B*) Confirmation of myocardial injury 2 days after ISOP treatment. Representative images of H&E and Sirius red stained slices, showing immune cell infiltration and early fibrotic changes, respectively. (*C*) Serum cTNI concentrations measured by enzyme-linked immunosorbent assay. *P < 0.05, Mann–Whitney *U* test, *n* = 6/group. (*D*) Left: Ejection fraction (EF) measured by echocardiography 2 days after ISOP or CON treatment. *P < 0.05, repeated measures ANOVA, Bonferroni’s post-hoc test, *n* = 6/group. Right: Representative parasternal long-axis and M-mode echocardiographic images 2 days after ISOP or CON treatment. (*E*) Kaplan–Meier curves of survival after ISOP and CON treatment. (*F*) EF and left ventricular remodelling index (LVRi, normalized to body surface area [BSA]) after 2 and 16 weeks of ISOP or CON treatment. *P < 0.05, mixed effects model, Tukey’s post-hoc test, *n* = 28–47/group. (*G*) Representative parasternal long-axis and M-mode echocardiographic images 16 weeks after ISOP or CON treatment.

Myocardial ischaemic injury was induced by intraperitoneal (i.p.) administration of 160 mg/kg isoprenaline (ISOP group) or its vehicle, phosphate-buffered saline (PBS) (control [CON] group), representing an established model of myocardial injury through Type 2 myocardial infarction (MI), as described previously.^19–22^ Altogether, 81 male C56Bl/6J mice were randomized after baseline echocardiography into ISOP (*n* = 43) or CON groups (*n* = 38, *Figure 1A*). To confirm acute ischaemic injury after ISOP treatment, six mice per group were randomly selected after 2 days of initial treatment for measurement of cardiac function via echocardiography and collection of heart and blood samples after termination of animals (*Figure 1B–D*). The remaining animals were followed up for 16 weeks post-myocardial ischaemic injury, with measurements of cardiac function on Weeks 2 and 16. Mice with abnormal cardiac function or morphology (e.g. severe LV dilation, severely decreased systolic function) at Week 16 were excluded from further analysis (*n* = 3 for CON group and *n* = 3 for ISOP group). After the follow-up period, mice from both groups were randomized into three further groups: Isotype, receiving 200 μg isotype control antibody i.p. (CON + Isotype: *n* = 9, ISOP + Isotype: *n* = 10); anti-PD-1 group, receiving 200 μg anti-PD-1 antibody i.p. (CON + anti-PD-1: *n* = 9, ISOP + anti-PD-1: *n* = 10); and anti-PD-1 + abatacept group, receiving 200 μg anti-PD-1 antibody i.p. combined with 200 μg abatacept i.p. (CON + ICI + abatacept: *n* = 8, ISOP + ICI + abatacept: *n* = 10). The randomization strategy involved minimizing initial group differences in cardiac function between the three subgroups. Anti-PD-1 antibody (Rat IgG2a, κ, clone RMP1-14, BP0146) and its isotype control (InVivoPlus rat IgG2a isotype control, clone 2A3, BP0089) were purchased from BioXcell, Lebanon, NH, USA. Abatacept was purchased from Bristol Myers Squibb Princeton, NJ, USA, and was supplied by the University Pharmacy of Semmelweis University. The dose of anti-PD-1 antibody was based on our group’s previous experiments,^23,24^ whereas the dose of abatacept was chosen according to pre-clinical studies showing its effect in improving cardiac dysfunction.^18,25^ Cardiac function was measured via echocardiography. Following this, mice were sacrificed under ketamine/xylazine anaesthesia (100/10 mg/kg) with cervical dislocation, followed by whole-body perfusion with PBS, after which organs were stored for histological and molecular analyses. Myocardial and systemic inflammatory response was measured via histology and immunohistochemistry, quantitative reverse transcription–polymerase chain reaction (qRT–PCR), and flow cytometry, as detailed below.

### 2.3 Echocardiography

Echocardiography was performed under isoflurane anaesthesia using a Vevo 3100 high-resolution in vivo imaging system (Fujifilm VisualSonics, Toronto, Canada) using an ultrahigh-frequency MX400 transducer (30 MHz, 55 frames per second), by an operator blinded to the study groups, as described previously.^26,27^ Mice were anaesthetized with isoflurane inhalation (5% for induction and 2% for maintenance). Echocardiographic recordings were evaluated with VevoLAB software (Fujifilm VisualSonics) by an evaluator blinded to the study groups. Detailed methods are provided in the Supplementary Methods.

### 2.4 Histology

Haematoxylin and eosin staining, Sirius red staining, and immunohistochemistry (with primary antibodies for PD-L1, Iba1, CD3ε, CD4, CD8, and Foxp3) were performed on myocardial samples. Detailed methods are provided in the Supplementary Methods.

### 2.5 RNA isolation and qRT–PCR

Total RNA was isolated from mouse heart samples with a chloroform/isopropanol precipitation method. cDNA was synthesized from 1 µg total RNA and qRT–PCR reactions were performed on a LightCycler® 480 II instrument (Roche, Germany). Sequences of primers are shown in Supplementary material online, *Table S1*. Ribosomal protein L13a (Rpl13a) was used as housekeeping gene. Results were calculated with 2-ΔΔCp evaluation method. Detailed methods are provided in the Supplementary Methods.

### 2.6 Enzyme-linked immunosorbent assay

The concentration of cardiac Troponin-I (cTNI) in the mouse serum samples was detected by Ultrasensitive Mouse Cardiac Troponin-I ELISA kit (CTNI-1-US, Life Diagnostics, West Chester, PA, USA) according to the manufacturer’s instructions. Detailed methods are provided in the Supplementary Methods.

### 2.7 Western blot

Western blot was performed on homogenized thymus samples with a primary antibody for PD-1. Detailed methods are provided in the Supplementary Methods.

### 2.8 Flow cytometry

Thymuses were homogenized and stained with primary antibodies against CD3, CD4, CD8, CD25, FOXP3, or isotype controls. The list of antibodies and isotype controls used are shown in Supplementary material online, *Table S2*. Detailed methods are provided in the Supplementary Methods.

### 2.9 Clinical study design, setting, and population

**Figure 2:**
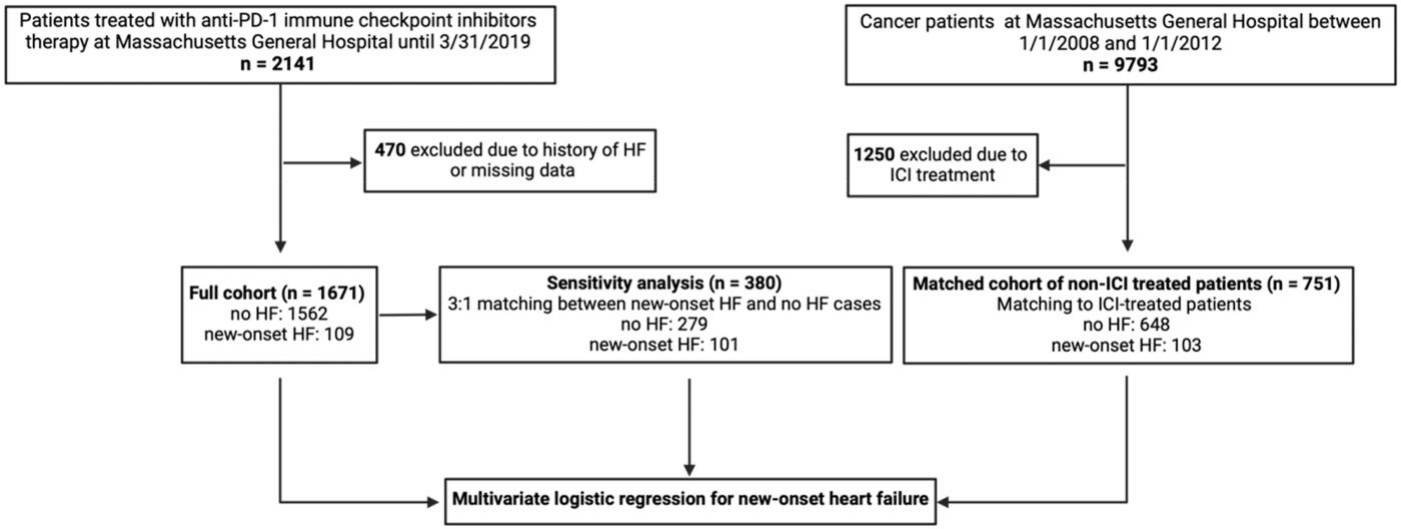
Flow diagram of the clinical study. In the sensitivity analysis, patients were matched for age, gender, cancer type, BMI, hypertension, diabetes, dyslipidaemia, and follow-up time. Non-ICI-treated cancer patients were matched to ICI-treated patients for age, gender, cancer type, BMI, hypertension, diabetes, dyslipidaemia, and follow-up time. ICI, immune checkpoint inhibitor; HF, heart failure.

For the clinical study, the Partners Human Research Committee approved the study, and no informed consent was required. The retrospective matched cohort study of patients treated with a PD-1 inhibitor was performed through the end of March 2019 at a single academic institution (Massachusetts General Hospital, Boston, MA, USA) (*n* = 2141). The design of the study is shown in *Figure 2*. The use of a PD-1 inhibitor was derived from a pharmacy database. Patients with combination ICI treatment were excluded from the study. The study entry date was defined as the first date a PD-1 inhibitor was administered. Covariates were derived from the Research Patient Data Registry. Patients with a history of heart failure or missing data on risk factors or follow-up were excluded, resulting in 1671 patients. Cases were defined as patients who developed incident heart failure after PD-1 inhibitor therapy started. Controls were patients who did not develop heart failure after ICI started. As a sensitivity analysis, patients with new-onset heart failure were matched with patients without heart failure development for age, gender, cancer type, body mass index (BMI), hypertension, diabetes, dyslipidaemia, and follow-up time.

Patients with cancer who did not receive ICI therapy were selected from all patients treated for cancer at our centre between 1 January 2008, and 31 December 2012. This cohort has been previously published.^4^ The use of an ICI at any time point was an exclusion criterion. The study entry for the controls was their first visit after 1 January 2008. Of these, non-ICI-treated cancer patients were matched for age, gender, cancer type, BMI, hypertension, diabetes, dyslipidaemia, and follow-up time to ICI-treated patients.

### 2.10 Clinical study procedures and clinical outcomes

Covariates of interest obtained included patient demographics, medications, standard cardiovascular risk factors (e.g. diabetes mellitus, hypertension, and smoking), vital parameters, and laboratory results. Data relevant to cancer included the cancer type and prior potentially cardiotoxic cancer therapies (radiation therapy, 5-fluorouracil, anthracyclines, and tyrosine kinase inhibitors). Data specific to the ICI cohort also included the number of ICI cycles, any immune-related adverse event occurrence, and the use of corticosteroids. The date of death was obtained from the RPDR. The authors vouch for the completeness and accuracy of the data and all analyses.

The primary outcome was incident heart failure during follow-up. Events were initially identified from the RPDR database using standard International Classification of Diseases (ICD)-9 and ICD-10 codes.

### 2.11 Statistical analysis

Values are presented as mean ± standard error of the mean for the pre-clinical experiments. For the clinical data, continuous variables are presented as mean (standard deviation) or median (25–75%). Categorical variables are presented as counts and percentages. Categorical data were compared with Pearson’s χ^2^ test or Fisher’s exact test. Normal distribution of the data was tested by Shapiro–Wilk normality test. For comparisons between two groups, either a parametric two-tailed Student’s *t-*test or a non-parametric Mann–Whitney *U* test was performed. The statistical analysis was performed using GraphPad Prism Software (version 8.0.1) and R studio software (version 1.4.1106). All statistical tests were two-tailed. *P* values of <0.05 were considered to indicate statistical significance.

In the pre-clinical study, repeated measures analysis of variance (ANOVA) followed by Bonferroni’s post-hoc test was used for multiple comparisons between related groups. One-way ANOVA followed by Tukey’s post-hoc test or Kruskal–Wallis test followed by Dunn’s post-hoc test was used to compare independent groups.

In the clinical study, univariable and multivariable logistic regression analysis was performed using the logit package. As a sensitivity analysis, 3:1 propensity score matching was performed using the MatchIt package, with a generalized linear model and calliper of 0.1 without replacement. The following variables were used to create propensity scores: age, gender, cancer type, BMI, hypertension, diabetes, dyslipidaemia, and follow-up time. Standardized mean differences were used to examine the balance of covariate distribution between the groups.

## 3. Results

### 3.1 Pre-clinical model of reversible cardiac ischaemic injury

To investigate the effect of prior cardiac ischaemic injury on immune checkpoint inhibition-related new-onset heart failure development, we induced reversible myocardial ischaemic injury in mice, with functional recovery during long-term follow-up. C57BL/6J mice were treated with ISOP (ISOP group) or PBS (CON group), representing a model of Type 2 MI,^19^ and were followed up for 16 weeks (*Figure 1A*). Altogether, 81 mice were treated (*n* = 38 in the CON group and *n* = 43 in the ISOP group).

Confirmation of myocardial ischaemic injury was performed on six randomly selected animals from each group 2 days after the injection. On haematoxylin and eosin-stained slices, immune cell infiltration was visible, while Sirius red staining showed early fibrotic changes in the myocardium of ISOP-treated animals (*Figure 1B*). cTNI release was significantly increased in the ISOP group (*Figure 1C*), indicating cardiomyocyte injury. Echocardiographic measurements showed decreased systolic function 2 days after ISOP treatment, while cardiac function was preserved in the CON group (*Figure 1D*).

Next, we aimed to confirm the successful functional recovery of mice during a 16-week follow-up period. Mortality occurred in the ISOP group up to 1 week after ISOP administration, while no animals died during the remaining follow-up period (*Figure 1E*). The systolic function of ISOP-treated mice was still mildly but significantly decreased after 2 weeks (*Figure 1F*), whereas successful functional recovery was observed at the end of the 16-week follow-up period, as seen by the normalized ejection fraction (EF) (*Figure 1F*) and global longitudinal strain (see Supplementary material online, *Figure S1*). Furthermore, the echocardiographic left ventricular remodelling index did not differ between groups, showing no significant cardiac remodelling (*Figure 1G*). Full echocardiographic comparisons between groups after the 16-week follow-up are shown in Supplementary material online, *Figure S1*.

In summary, reversible ischaemic injury was seen after ISOP treatment in C57BL/6J mice with initial immune infiltration and ischaemic injury, while successful functional recovery was observed after 16 weeks of follow-up.

### 3.2 Mice with prior cardiac ischaemic injury exhibit more severe cardiac dysfunction after anti-PD-1 treatment, and it is prevented by abatacept cotreatment

Next, we investigated whether prior cardiac ischaemic injury exacerbates cardiac dysfunction after anti-PD-1 treatment. Furthermore, we tested whether abatacept coadministration can prevent cardiac decline due to immune checkpoint inhibition.

**Figure 3:**
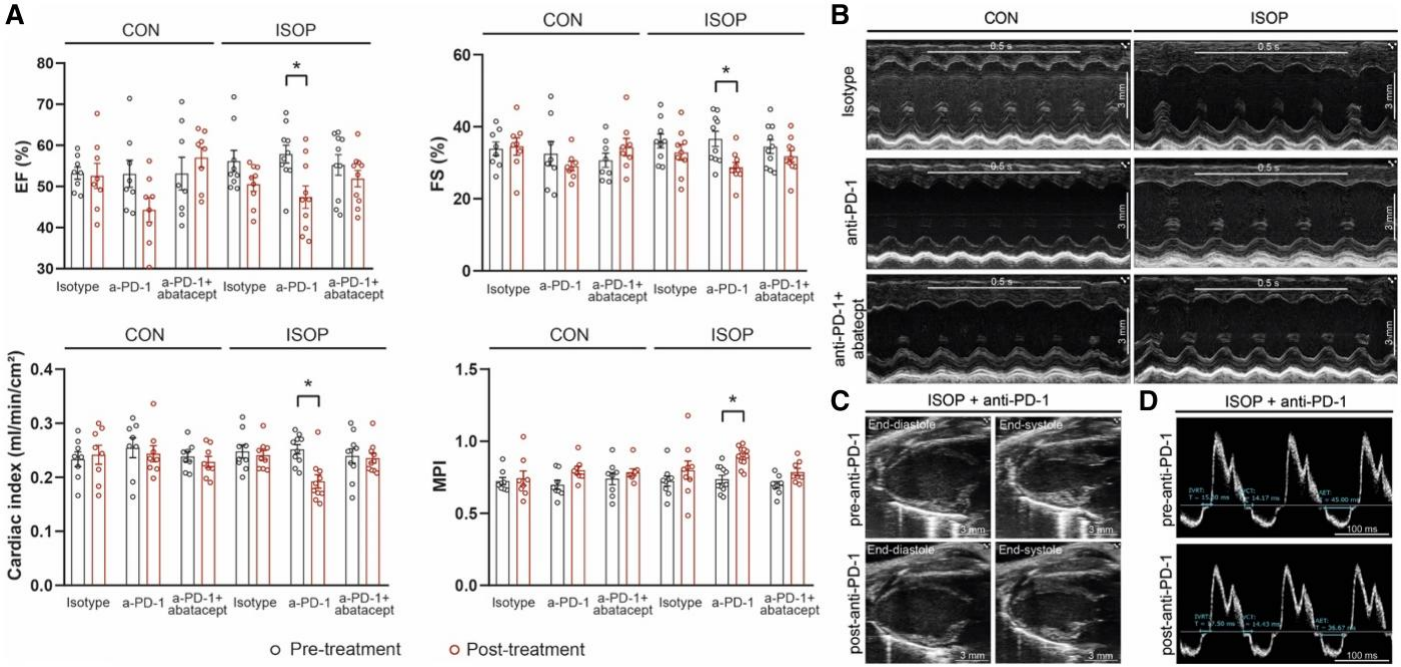
Assessment of cardiac function via echocardiography. (*A*) EF, FS, CI, and MPI (Tei index) before and after isotype control, anti-PD-1, or anti-PD-1 + abatacept treatment. *P < 0.05, repeated measures two-way ANOVA, Bonferroni’s post-hoc test, *n* = 8/group for CON groups, *n* = 10 for ISOP groups. (*B*) Representative M-mode echocardiographic images. (*C*) Representative parasternal long-axis echocardiographic images before and after anti-PD-1 treatment in mice with prior ischaemic injury (ISOP group). (*D*) Representative pulse-wave Doppler images before and after anti-PD-1 treatment in mice with prior ischaemic injury (ISOP group).

During the 2-week treatment period, no animals died. Markers of left ventricular systolic function, i.e. EF and fractional shortening (FS), significantly decreased after anti-PD-1 administration in mice with prior ischaemic injury (ISOP + anti-PD-1), compared with the corresponding baseline measurements (*Figure 3A*, representative images: *Figure 3B* and *C*). Cardiac index (CI) decreased significantly in the ISOP + anti-PD-1 group, compared with pre-treatment levels, while heart rate was unaffected (see Supplementary material online, *Figure S2*). Myocardial performance index (MPI or Tei index) increased in ISOP + anti-PD-1 treated animals compared with its respective baseline value, which was mainly due to the shortening of ejection time (representative image: *Figure 3D*). In animals without ischaemic injury (CON group), anti-PD-1 treatment led to a non-significant decrease in cardiac systolic function measured by EF (*P* = 0.07). Importantly, no significant alteration was observed in any of the functional parameters (EF, FS, CI, and MPI) when abatacept was coadministered with anti-PD-1 treatment, even in mice with prior cardiac ischaemic injury (*Figure 3A*). Diastolic function, as assessed by the ratio of early diastolic filling and early diastolic mitral annular tissue velocity (E/e′) or isovolumetric relaxation time, was not affected by ICI treatment in any group (see Supplementary material online, *Figure S2*).

Altogether, anti-PD-1 treatment led to exacerbated cardiac systolic dysfunction in animals with prior ischaemic injury, while worsening of cardiac function could be prevented by the coadministration of abatacept.

### 3.3 Cardiac inflammation is exacerbated in mice with prior cardiac ischaemic injury after anti-PD-1 treatment, and it is alleviated by abatacept cotreatment

**Figure 4:**
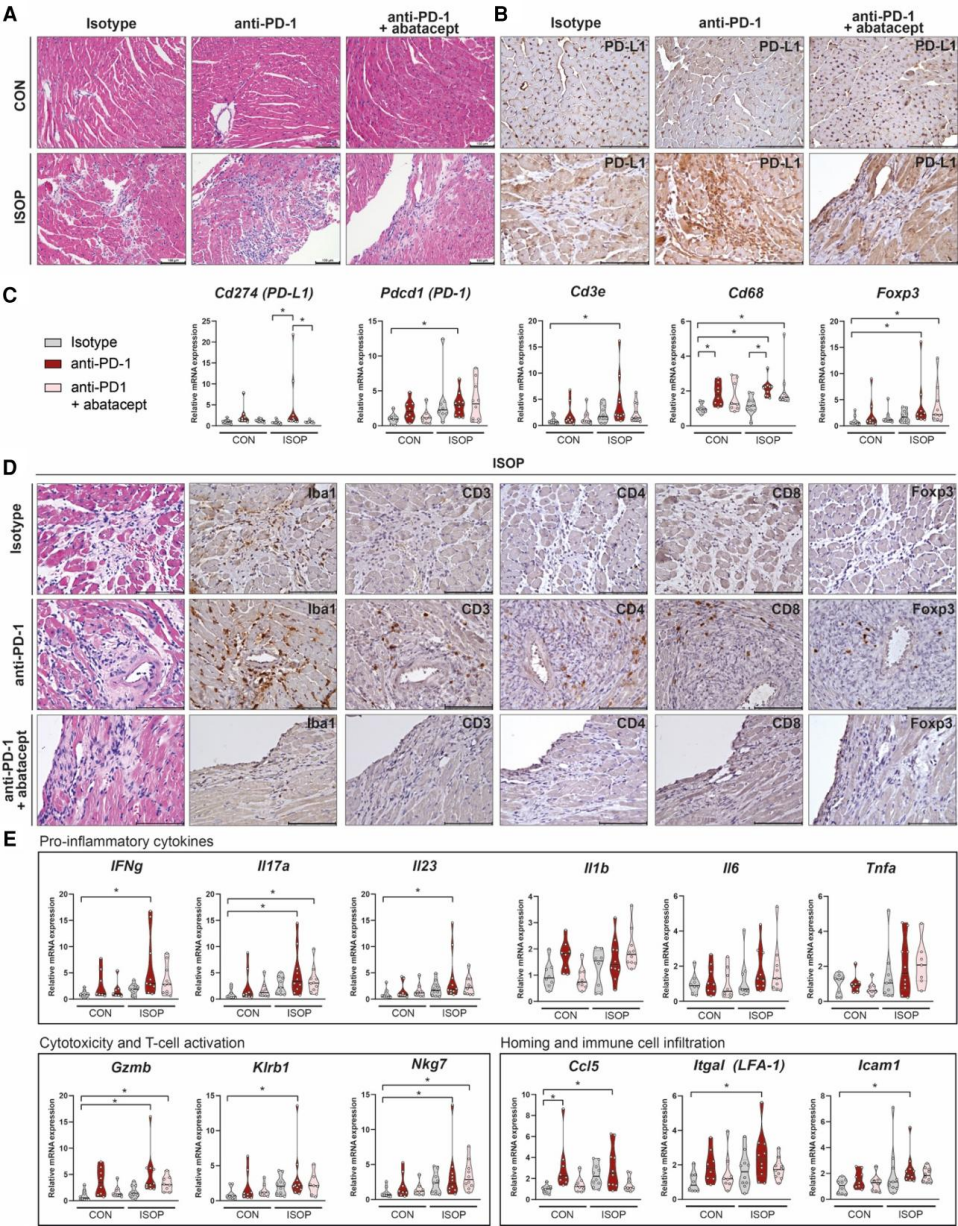
Assessment of myocardial inflammation after immune checkpoint inhibition. (*A*) Representative images of H&E-stained slices. (*B*) Representative images of PD-L1 immunohistochemistry. (*C*) Quantification of immune checkpoint expression (CD274, Pdcd1) and cellular markers (Cd3e, Cd68, and Foxp3) via qRT–PCR. *P < 0.05, Kruskal–Wallis test, followed by Dunn’s post-hoc test, *n* = 8/group for CON groups, *n* = 10 for ISOP groups. (*D*) Representative images of selected serial myocardial lesions from ISOP-treated animals stained via immunohistochemistry for cellular markers. (*E*) Quantification of cytokine and chemokine mRNA expressions via qRT–PCR. *P < 0.05, Kruskal–Wallis test, followed by Dunn’s post-hoc test, *n* = 8/group for CON groups, *n* = 10 for ISOP groups.

In the CON group, myocardial immune cell infiltrations were not observed on haematoxylin and eosin staining in any mice, including animals treated with anti-PD-1 (*Figure 4A*). Mice with prior ischaemic injury (ISOP group) treated with isotype control antibody showed fibrotic lesions with mild immune cell infiltration, as signs of the previous ischaemic injury. Significant immune cell infiltrations were seen in animals treated with anti-PD-1 with prior ischaemic injury (ISOP + ICI group), while the anti-PD-1 + abatacept cotreated groups were similar to the isotype-treated animals in terms of infiltrations (*Figure 4A*).

A key histological marker of ICI-induced inflammation is PD-L1 expression in the myocardium,^28^ on infiltrating immune cells and endothelial cells, presumably as a negative feedback mechanism. PD-L1 immunohistochemistry have revealed high-intensity PD-L1 staining of infiltrating immune cells and cardiac vasculature in the anti-PD-1 treated animals with prior ischaemic injury (ISOP + ICI), which was not observed in other groups (*Figure 4B*). Upon quantification with qRT–PCR, an increase in *Cd274* (encoding PD-L1) expression was seen in the ISOP + ICI group compared with both ISOP + Isotype and ISOP + ICI + abatacept groups (*Figure 4C*). Furthermore, *Pdcd1* (encoding PD-1) expression was higher in the ISOP + anti-PD-1 group (*Figure 4C*). The T-cell marker *Cd3e* was increased only in the ISOP + anti-PD-1 group compared with CON animals, while *Foxp3*, a marker of regulatory T cells, was increased in ISOP + anti-PD-1 and ISOP + anti-PD-1 + abatacept groups (*Figure 4C*). Interestingly, macrophage marker *Cd68* was elevated in anti-PD-1-treated animals regardless of ischaemic injury (*Figure 4C*), suggesting that prior ischaemic injury mainly contributes to T-cell infiltration after ICI treatment.

Next, we characterized the cellular composition of the immune infiltrates in the ISOP-treated groups. We have performed immunohistochemistry for several immune markers on the same infiltrative lesions (*Figure 4D*). While Iba1^+^ macrophages were present in all lesions, the presence of CD3^+^ T cells only occurred in anti-PD-1-treated animals, while CD3^+^ T cells were not observed after abatacept administration. Furthermore, CD4^+^, CD8^+^, and Foxp3^+^ T-cell subsets were present in the myocardium of animals with prior ischaemic injury and anti-PD-1 treatment, while lacking in mice cotreated with abatacept (*Figure 4D*).

We also analysed the expression of a panel of T-cell-related pro-inflammatory cytokines in the myocardium with qRT–PCR. We found increased *Il17a*, *Il23*, and *Ifng,* pro-inflammatory cytokine expression in the ISOP + ICI group, while other pro-inflammatory cytokines did not show significant differences, including *Il1b*, *Il6,* or *Tnfa* (*Figure 4E*). Markers of T-cell-mediated cytotoxicity, such as *Gzmb*, *Klrb1*, and *Nkg7*, were also increased after anti-PD-1 treatment in the ISOP-treated animals. Cotreatment with abatacept prevented the increase in pro-inflammatory markers, except for *Il17a*, *Gzmb,* and *Nkg7*. *Ccl5*, a marker of immune cell homing, was increased after anti-PD-1 treatment regardless of prior ischaemic injury (*Figure 4E*), while further markers of immune cell infiltration, like *Itgal* and *Icam1*, were increased only in the ISOP + anti-PD-1 group.

Next, we investigated further hallmarks of ICI-induced myocarditis, previously reported in pre-clinical models.^15,29–31^ We found that serum levels of cTNI were not significantly different among groups (see Supplementary material online, *Figure S3*). Similarly, on electrocardiogram measurements after 2 weeks of treatment, arrhythmias were not observed, although a slight increase in QRS duration was visible (see Supplementary material online, *Figure S4*). On haematoxylin and eosin staining, no visible cardiomyocyte loss was observed, corresponding to myocardial inflammation but no definitive myocarditis based on the pathological classification proposed by Palaskas *et al.*^28^

Altogether, myocardial inflammation was increased in animals with prior ischaemic injury treated with anti-PD-1. This finding was mostly due to increased T-cell infiltration and activation, while cotreatment with abatacept alleviated the pro-inflammatory effects. Nevertheless, the absence of cardiomyocyte damage, arrhythmias, and lack of mortality was not consistent with previously reported models of fulminant ICI-myocarditis, but rather represented a moderate level of inflammation in the myocardium associated with cardiac dysfunction.

### 3.4 Systemic immune response after immune checkpoint modulation: thymus alterations

**Figure 5:**
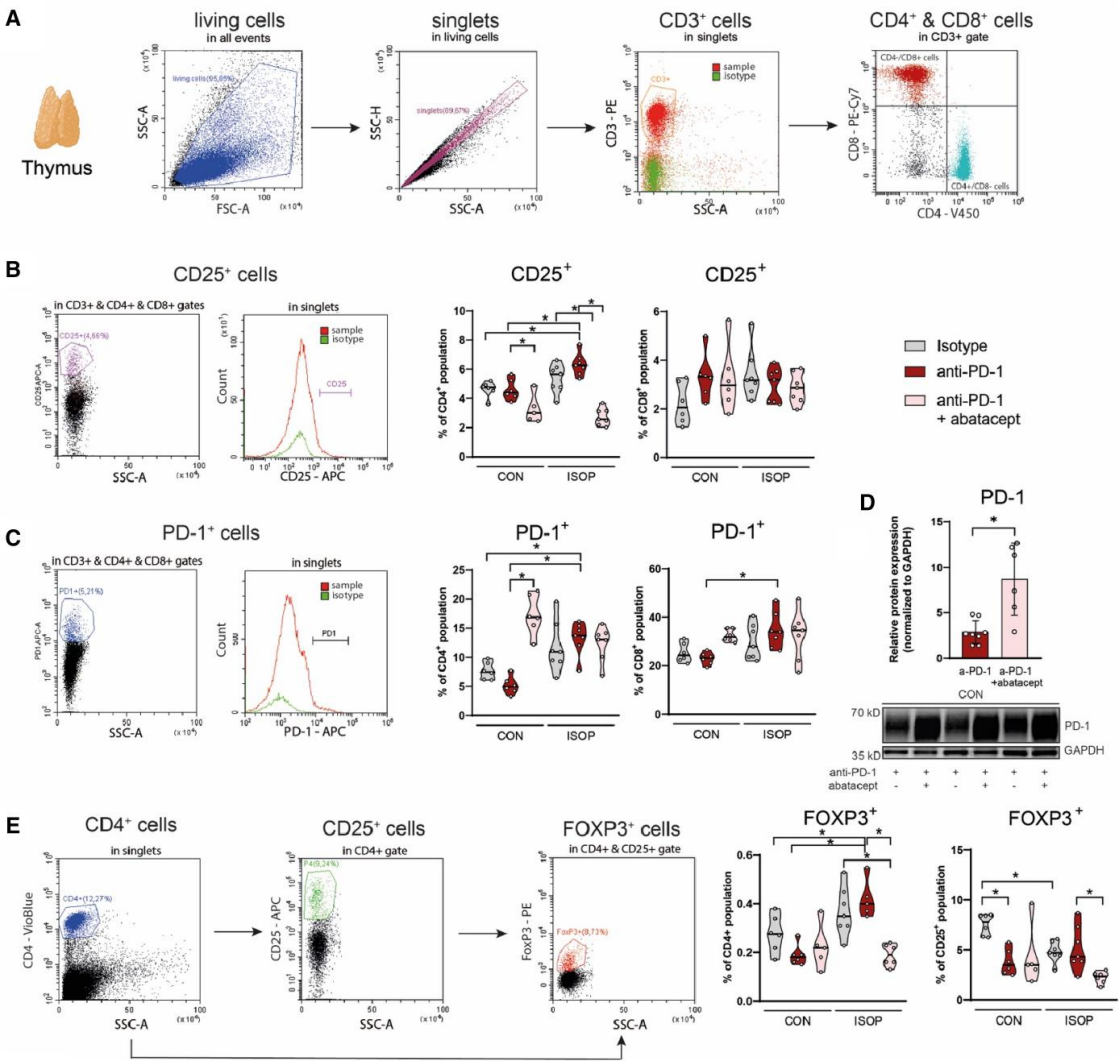
Assessment of immunological changes in the thymus via flow cytometry. (*A*) Gating strategy for CD4^+^ and CD8^+^ cells. (*B*) Gating strategy and quantification of CD25^+^ cells in CD4^+^ and CD8^+^ populations. *P < 0.05, Kruskal–Wallis test, followed by Dunn’s post-hoc test, *n* = 5–6/group for CON groups, *n* = 7 for ISOP groups. (*D*) Gating strategy and quantification of PD-1^+^ cells in CD4^+^ and CD8^+^ populations. *P < 0.05, Kruskal–Wallis test, followed by Dunn’s post-hoc test, *n* = 5–6/group for CON groups, *n* = 7 for ISOP groups. (*D*) Quantification of PD-1 expression in via western blot. *P < 0.05, Student’s *t*-test. (*E*) Gating strategy and quantification of FOXP3^+^ cells in CD4^+^ and CD8^+^ populations. *P < 0.05, Kruskal–Wallis test, followed by Dunn’s post-hoc test, *n* = 5–6/group for CON groups, *n* = 7 for ISOP groups.

Next, we aimed to investigate systemic inflammatory response to ICI treatment with or without previous ischaemic injury. Thymus alterations have been associated with susceptibility to ICI-myocarditis previously,^32^ while pro-inflammatory cytokine upregulation in the thymus has also been shown to be involved in ICI-induced cardiac dysfunction.^23,33^ Here, we investigated markers of T-cell activation in the thymus that either promote (CD25) or suppress (PD-1, FOXP3) the subsequent immune response with flow cytometry. We found that the number of CD25^+^ CD4^+^ T cells increased in ISOP + anti-PD-1 treated animals compared to control mice, while a decrease was observed after abatacept cotreatment with or without prior ischaemic injury, with similar findings observed in the case of FOXP3^+^ cells (*Figure 5A, B,* and *E*). Interestingly, the number of PD1^+^ T_reg_ cells in the thymus was decreased after anti-PD-1 treatment in additional experiments (see Supplementary material online, *Figure S5*). In mice without ischaemic injury, the addition of abatacept increased the number of PD-1^+^ CD4^+^ T cells (*Figure 5C*), which effect was confirmed by Western blot analysis showing increased PD-1 protein expression (*Figure 5D*), suggesting a potential negative regulatory effect of abatacept. Interestingly, in mice with prior ischaemic injury, the number of PD-1^+^ CD4^+^ and CD8^+^ cells were increased after anti-PD-1 treatment, which was not altered by abatacept cotreatment, suggesting that negative feedback mechanisms are occurring in mice with prior ischaemic injury and ICI-related cardiotoxicity, not affected by abatacept.

### 3.5 Prior cardiac ischaemic events are associated with new-onset heart failure in patients after ICI therapy

#### 3.5.1 Full cohort

Out of the 1671 patients treated with ICI, during a median follow-up time of 332.0 days [inter-quartile range (IQR): 93.0–827.5], 109 (6.5%) patients developed heart failure (*Table 1*). Patients with new-onset heart failure had higher median age and BMI, while there was no significant difference between the sexes of the patients. Of the cardiovascular risk factors, prior hypertension, diabetes, and dyslipidaemia were more frequent in the heart failure group. Prior myocardial ischaemic events [defined as a history of either MI, percutaneous coronary intervention (PCI), or coronary artery bypass graft surgery (CABG)] were significantly higher in patients with new-onset heart failure after ICI initiation compared to controls (17 vs. 5.6%, respectively). There were no significant differences between the concomitant use of other standard cardiotoxic anti-cancer drugs (e.g. anthracyclines, tyrosine kinase inhibitors, platinum analogues, 5-fluorouracil). There was a longer follow-up time among those who developed heart failure (median 556.0, IQR: 238.0–1116.0 days) compared with those who did not (median 307.0, IQR: 84.0–804.8 days) (*P* < 0.001). In a multivariable logistic regression model accounting for age, gender, BMI, hypertension, diabetes, dyslipidaemia, renal cancer, and follow-up time, prior myocardial ischaemic events remained significantly associated with new-onset heart failure development with an odds ratio (OR) of 2.34 [95% confidence interval (CI) 1.26–4.16, *P* = 0.005, *Table 2*].

**Table 1:** Baseline characteristics of the full cohort and the sensitivity analysis cohort (with 3:1 matching)

	Full cohort of patients receiving ICI				Sensitivity analysis (3:1 matched)			
		No HF	New HF			No HF	New HF	
	*n*	*n* = 1562	*n* = 109	*P*-value	*n*	*n* = 279	*n* = 101	*P*-value
Demographics								
Gender, no. (%)	1671			0.078	380			0.6
Female		709 (45%)	40 (37%)			110 (39%)	37 (37%)	
Male		853 (55%)	69 (63%)			169 (61%)	64 (63%)	
Age, year, median (IQR)	1671	65 (56–72)	68 (62–75)	**<0.001**	380	69 (61–75)	69 (62–75)	0.9
Race, no. (%)	1627			0.6	374			0.5
Asian		63 (4.2%)	5 (4.6%)			9 (3.3%)	4 (4.0%)	
Black or African American		33 (2.2%)	4 (3.7%)			4 (1.5%)	4 (4.0%)	
Other		46 (3.0%)	2 (1.8%)			7 (2.6%)	2 (2.0%)	
White		1376 (91%)	98 (90%)			253 (93%)	91 (90%)	
Clinical variables, mean (SD)								
BMI (kg/m^2^)	1605	25.5 (22.3, 29.2)	27.8 (23.6, 31.0)	**0**.**003**	380	26.5 (23.7, 30.7)	27.4 (23.6, 30.3)	>0.9
Systolic blood pressure (mmHg)	1586	125 (113, 138)	131 (114, 146)	0.053	376	127 (117, 139)	134 (115, 146)	0.2
ICI therapy characteristics								
ICI type, no. (%)	1671			0.5	380			0.5
Nivolumab		655 (42%)	42 (39%)			122 (44%)	40 (40%)	
Pembrolizumab		907 (58%)	67 (61%)			157 (56%)	61 (60%)	
ICI cycles, no (IQR)	1671	5 (2–14)	7 (3–16)	0.071	380	9 (3–20)	7 (316)	0.15
Cancer type, no. (%)	1671				380			
Brain		11 (0.7%)	0 (0%)			1 (0.4%)	0 (0%)	
Breast		77 (4.9%)	4 (3.7%)			19 (6.8%)	4 (4.0%)	
Gastrointestinal		54 (3.5%)	1 (0.9%)			7 (2.5%)	1 (1.0%)	
Head and neck		229 (15%)	13 (12%)			32 (11%)	12 (12%)	
Hepatobiliary		62 (4.0%)	2 (1.8%)			6 (2.2%)	2 (2.0%)	
Hodgkin’s		15 (1.0%)	1 (0.9%)			2 (0.7%)	1 (1.0%)	
Lung		492 (31%)	36 (33%)			86 (31%)	33 (33%)	
Lymphoma		40 (2.6%)	4 (3.7%)			6 (2.2%)	4 (4.0%)	
Melanoma		351 (22%)	25 (23%)			67 (24%)	23 (23%)	
Gynaecologic		73 (4.7%)	4 (3.7%)			20 (7.2%)	3 (3.0%)	
Pancreatic		11 (0.7%)	1 (0.9%)			0 (0%)	1 (1.0%)	
Renal		88 (5.6%)	11 (10%)			22 (7.9%)	11 (11%)	
Other		59 (3.8%)	7 (6.4%)			11 (3.9%)	6 (5.9%)	
Cardiovascular risk factors and comorbidities, no. (%)								
Arrhythmia	1671	568 (36%)	57 (52%)	**<0**.**001**	380	101 (36%)	56 (55%)	**<0**.**001**
Chronic pulmonary disease	1671	489 (31%)	48 (44%)	**0**.**006**	380	88 (32%)	46 (46%)	**0**.**012**
Diabetes	1671	162 (10%)	19 (17%)	**0**.**022**	380	39 (14%)	17 (17%)	0.5
Chronic kidney disease	1671	151 (9.7%)	22 (20%)	**<0**.**001**	380	35 (13%)	22 (22%)	**0**.**026**
Hyperlipidaemia	1671	687 (44%)	69 (63%)	**<0**.**001**	380	179 (64%)	63 (62%)	0.7
Smoking	1671	584 (37%)	40 (37%)	0.9	380	106 (38%)	38 (38%)	>0.9
Dyslipidaemia	1668	437 (28%)	52 (48%)	**<0**.**001**	380	122 (44%)	48 (48%)	0.5
Prior cardiovascular events, no. (%)								
History of CABG	1671	21 (1.3%)	6 (5.5%)	**0**.**006**	380	6 (2.2%)	6 (5.9%)	0.091
History of PCI	1671	44 (2.8%)	6 (5.5%)	0.13	380	13 (4.7%)	6 (5.9%)	0.6
History of any coronary revascularization	1671	65 (4.2%)	10 (9.2%)	**0**.**027**	380	19 (6.8%)	10 (9.9%)	0.3
History of MI	1671	51 (3.3%)	13 (12%)	**<0**.**001**	380	14 (5.0%)	13 (13%)	**0**.**008**
Prior cardiac ischaemic event (MI, PCI, or CABG)	**1671**	**87** (**5.6%)**	**18** (**17%)**	**<0**.**001**	**380**	**26** (**9.3%)**	**18** (**18%)**	**0**.**022**
Stroke	1671	38 (2.4%)	4 (3.7%)	0.3	380	4 (1.4%)	4 (4.0%)	0.2
Any CV event	1671	123 (7.9%)	20 (18%)	**<0**.**001**	380	30 (11%)	20 (20%)	**0**.**021**
Prior anti-cancer therapy, no. (%)								
Anthracycline	1671	97 (6.2%)	7 (6.4%)	>0.9	380	19 (6.8%)	7 (6.9%)	>0.9
5-Fluorouracil	1671	176 (11%)	8 (7.3%)	0.2	380	27 (9.7%)	7 (6.9%)	0.4
Platinum	1671	717 (46%)	44 (40%)	0.3	380	129 (46%)	42 (42%)	0.4
Tyrosine kinase inhibitor	1671	78 (5.0%)	9 (8.3%)	0.14	380	11 (3.9%)	8 (7.9%)	0.12
Baseline cardiovascular medications, no. (%)								
Angiotensin-converting-enzyme inhibitors	1671	379 (24%)	45 (41%)	**<0**.**001**	380	95 (34%)	42 (42%)	0.2
Angiotensin receptor blocker	1671	137 (8.8%)	12 (11%)	0.4	380	35 (13%)	11 (11%)	0.7
Beta-blocker	1671	533 (34%)	53 (49%)	**0**.**002**	380	125 (45%)	51 (50%)	0.3
Calcium channel blocker	1671	317 (20%)	44 (40%)	**<0**.**001**	380	79 (28%)	41 (41%)	**0**.**023**
Diuretics	1671	403 (26%)	37 (34%)	0.062	380	89 (32%)	34 (34%)	0.7
Statin	1671	562 (36%)	57 (52%)	**<0**.**001**	380	150 (54%)	52 (51%)	0.7
Anti-arrhythmic drugs	1671	97 (6.2%)	12 (11%)	**0**.**05**	380	21 (7.5%)	12 (12%)	0.2
Antiplatelet drugs	1671	48 (3.1%)	8 (7.3%)	**0**.**026**	380	17 (6.1%)	7 (6.9%)	0.8
Aspirin	1671	543 (35%)	62 (57%)	**<0**.**001**	380	129 (46%)	58 (57%)	0.054
Low molecular weight heparin	1671	281 (18%)	17 (16%)	0.5	380	50 (18%)	16 (16%)	0.6
Novel oral anticoagulant	1671	72 (4.6%)	15 (14%)	**<0**.**001**	380	14 (5.0%)	14 (14%)	**0**.**004**
Warfarin	1671	79 (5.1%)	10 (9.2%)	0.064	380	21 (7.5%)	10 (9.9%)	0.5
Immune-related adverse events, no. (%)								
Any adverse events	1667	577 (37%)	61 (56%)	**<0**.**001**	380	124 (44%)	57 (56%)	**0**.**039**
Gastrointestinal	1667	232 (15%)	24 (22%)	**0**.**046**	380	55 (20%)	24 (24%)	0.4
Skin	1667	213 (14%)	28 (26%)	**<0**.**001**	380	50 (18%)	25 (25%)	0.14
Pneumonitis	1667	89 (5.7%)	11 (10%)	0.089	380	22 (7.9%)	10 (9.9%)	0.53
Hepatitis	1667	86 (5.5%)	6 (5.5%)	>0.9	380	13 (4.7%)	6 (5.9%)	0.6
Endocrine	1667	68 (4.4%)	4 (3.7%)	>0.9	380	13 (4.7%)	3 (3.0%)	0.6
Nephritis	1667	38 (2.4%)	5 (4.6%)	0.2	380	5 (1.8%)	5 (5.0%)	0.14
Neuromuscular	1645	36 (2.3%)	6 (5.5%)	0.055	375	14 (5.1%)	5 (5.0%)	>0.9
Pancreatitis	1667	22 (1.4%)	3 (2.8%)	0.2	380	3 (1.1%)	3 (3.0%)	0.2
Corticosteroid use for irAEs	1514	356 (25%)	37 (35%)	**0**.**035**	362	79 (30%)	34 (34%)	0.4

Statistics: Categorical data were compared with Pearson’s χ^2^ test or Fisher’s exact test. Differences in continuous variables were evaluated with the Mann–Whitney *U* test or Student’s *t*-test as appropriate. Significant *P*-values are marked in bold.

CV, cardiovascular; ICI, immune checkpoint inhibitor; HF, heart failure; SD, standard deviation.

**Table 2:** Multivariable logistic regression for new-onset heart failure

	Full cohort			Sensitivity analysis (3:1 matched)			Non-ICI-treated cancer patients		
Variables	OR	95% CI	*P*-value	OR	95% CI	*P*-value	OR	95% CI	*P*-value
Age	1.02	1.00–1.04	**0**.**021**	1.00	0.97–1.02	0.8	1.03	1.01–1.05	**0**.**002**
Female	0.85	0.55–1.29	0.4	0.96	0.58–1.56	0.9	0.89	0.57–1.38	0.6
BMI	1.04	1.01–1.07	**0**.**005**	0.99	0.95–1.03	0.6	0.99	0.95–1.04	0.7
Hypertension	1.35	0.83–2.22	0.2	1.10	0.63–1.95	0.7	1.74	1.07–2.87	**0**.**027**
Diabetes	1.36	0.77–2.31	0.3	1.26	0.65–2.39	0.5	1.24	0.63–2.28	0.5
Dyslipidaemia	1.45	0.93–2.26	0.1	0.99	0.60–1.63	>0.9	1.08	0.47–2.30	0.9
Prior MI/PCI/CABG	2.34	1.26–4.16	**0**.**005**	2.11	1.05–4.2	**0**.**033**	1.23	0.53–2.63	0.6
Renal cancer	1.75	0.84–3.37	0.11	1.42	0.63–3.04	0.4	1.04	0.40–2.39	0.9
Follow-up time (days)	1.00	1.00–1.00	**<0**.**001**	1.00	1.00–1.00	0.7	1.00	1.00–1.00	**<0**.**001**

Statistics: Multivariate logistic regression. Significant *P*-values are marked in bold.

BMI, body mass index; CI, confidence interval; CV, cardiovascular; ICI, immune checkpoint inhibitor; HF, heart failure; OR, odds ratio; SD, standard deviation.

#### 3.5.2 Sensitivity analysis for the matched cohort

In a sensitivity analysis, patients were matched for age, gender, cancer type, BMI, hypertension, diabetes, dyslipidaemia, and follow-up time, with a median follow-up of 596.0 days (IQR: 194.5–1082.5). In the subgroup analysis, the median follow-up time was not different among those who developed heart failure and those who did not [564 (IQR: 233.0–1124.0) vs. 615.0 (IQR: 166.0–1029.5) days, *P* = 0.48, *Table 1*]. A multivariable logistic regression was performed, and the odds ratio for future heart failure was 2.11 (95% CI 1.09–4.02, *P* = 0.024) with a history of prior ischaemic cardiac events (*Table 2*).

#### 3.5.3 Matched non-ICI-treated cancer patients

Historical controls (cancer patients who did not receive ICI therapy) were matched for age, gender, cancer type, BMI, hypertension, diabetes, dyslipidaemia, and follow-up time. Baseline characteristics of the matched cancer patient cohort (*n* = 751) are shown in Supplementary material online, *Table S3*. In this cohort, during a median follow-up time of 575 days (IQR: 186.5, 1063.0), 103 patients (13.7%) developed heart failure. Multivariable logistic regression found an association between hypertension and incident heart failure (OR 1.74, 95% CI 1.07–2.87, *P* = 0.027), but no statistically significant association between prior ischaemic events and incident heart failure (OR 1.23, 95% CI 0.53–2.63, *P* = 0.61).

Altogether, these findings suggest a potential significant interaction between prior cardiac ischaemic event–related myocardial injury and ICI use in new-onset heart failure development.

## 4. Discussion

In this study, among mice with previous ischaemic cardiac injury, the administration of a PD-1 inhibitor was associated with cardiac dysfunction and inflammation, adverse effects that were ameliorated by concomitant abatacept use. In a parallel clinical study, prior cardiac ischaemic events were associated with an increased risk for new-onset heart failure in cancer patients receiving ICI therapy (*Graphical Abstract*).

Cardiac dysfunction or heart failure without fulminant myocarditis is an increasingly recognized adverse effect of ICI therapy.^8,10,12,34–36^ Prospective cohort studies using cardiac magnetic resonance imaging have shown a small but significant decrease in EF across unselected patients receiving ICI therapy.^37^ However, little is known about its mechanisms or predisposing factors. Pre-existing cardiovascular disease is a potential risk factor for ICI-induced cardiotoxicity, as some studies showed that prior heart conditions predispose to major adverse cardiovascular events after ICI treatment,^38^ although previous observational studies have shown an inconsistent association between cardiovascular disease history and myocarditis.^6,39–42^ To investigate the effect of prior cardiac ischaemic injury on the development of heart failure after immune checkpoint inhibition, in our pre-clinical study, we used a reversible cardiac ischaemic injury model in mice. To ensure that we are solely investigating the effect of prior ischaemic injury, mice were allowed to fully recover after the injury, which was confirmed by echocardiography 16 weeks later. After 2 weeks of treatment with monoclonal antibodies, cardiac systolic function was most decreased in mice with previous ischaemic injury who were then treated with an anti-PD-1 antibody. Moreover, in our complementary retrospective cohort of cancer patients treated with ICI, prior cardiac ischaemic events were associated with new-onset heart failure development. The overall 6.5% rate of new-onset heart failure after ICI treatment is in concordance with previous retrospective and prospective studies.^43,44^ ICIs are known to enhance the progression of atherosclerotic cardiovascular disease (ASCVD), leading to ischaemic cardiac events.^4^ However, the association between new-onset heart failure and prior ischaemic events remained significant after adjusting for risk factors of ASCVD (e.g. age, sex, hypertension, diabetes mellitus, dyslipidaemia), suggesting that myocardial ischaemic injury *per se* is an exacerbating factor of ICI-induced heart failure development. In a matched cohort of non-ICI-treated cancer patients, prior ischaemic events showed a weaker association with incident heart failure during the observed period compared to the ICI cohort. Thus, a previous ischaemic injury may increase the susceptibility to cardiac effects after ICI treatment in cancer patients, akin to hidden drug-induced cardiotoxicity.^45^

While the mechanisms of ICI-induced myocarditis are increasingly understood,^29,31,32,46^ little is known about the development of cardiac dysfunction without fulminant myocarditis.^47^ Previously, mild cardiac inflammation with metabolic alterations has been associated with early cardiac dysfunction after ICI treatment without overt myocarditis.^11^ Conversely, autoimmunity and infiltration of cardiotropic CD4^+^ T cells have been shown previously to be involved in heart failure pathogenesis.^48^ In our pre-clinical study, cardiac dysfunction development was accompanied by modestly increased immune cell infiltration. Interestingly, while macrophage infiltration was generally observed after ischaemic injury in all groups of mice, T-cell infiltration was most prominently seen in mice with previous ischaemic injury and anti-PD-1 treatment. In this group, the expression of molecules regulating immune cell infiltration (e.g. *Itgal*, *Icam1*) was upregulated, suggesting that prior ischaemic injury potentiates myocardial T-cell infiltration during ICI treatment. Moreover, infiltrated T cells were shown to be activated by the upregulation of pro-inflammatory cytokines (*Ifng*, *Il17a*, *Il23*) and markers of cytotoxicity (*Gzmb, Klrb1, Nkg7*). Recently, cardiac ischaemic injury was shown to induce the recruitment of myosin-specific tissue-resident memory T cells in the myocardium and increase the susceptibility to myocarditis upon PD-1 blockade.^14^ Nevertheless, clinically, ICI-induced myocarditis presents without systolic dysfunction in 50% of cases,^6,49^ suggesting that the mechanisms may not overlap entirely between myocarditis and heart failure development. In our model, the lack of mortality, cell death, arrhythmias, and cTN release suggested that the observed effects were independent of fulminant myocarditis.^31,46^ Thus, myocardial inflammation in our model was likely associated with cardiac dysfunction similar to heart failure with reduced EF, including T-cell infiltration^50^ and elevated levels of IL-17.^51^

Previous myocardial injury may predispose the heart to increased susceptibility to immune checkpoint blockade. In a pre-clinical study of PD-1^−/−^ mice, pre-treatment with low doses of ISOP followed by one high dose resulted in severely exacerbated myocardial injury, with the expansion of autoreactive CD8^+^ T cells in the myocardium and increased numbers of effector memory CD8^+^ T cells in the mediastinal lymph nodes.^22^ Based on this, the PD-1 axis seems to have a vital role in maintaining homeostasis and regulating inflammation in the myocardium, whereas inhibition of PD-1 signalling could result in enhanced cardiac inflammation, pro-inflammatory cytokine response, and subsequent cardiac dysfunction. Moreover, the threshold for cardiac dysfunction after inhibition of immune checkpoints may be altered by previous myocardial ischaemic injury.^10^ T_reg_ cells expressing PD-1 could be potential mediators of the inflammation threshold in the myocardium.^13^ In our experiments, Foxp3 expression in the heart and the number of Foxp3^+^ T_reg_ cells in the thymus increased in response to anti-PD-1 treatment in mice with prior ischaemic injury. However, PD-1 expression on thymic T_reg_ cells was decreased after anti-PD-1 treatment, suggesting a potential explanation for the observed pro-inflammatory effects. Other mechanisms for the increased toxicity seen in previous cardiac injury may include alterations of immune checkpoint signalling after MI^52^ and cardiac autoantibody development,^53,54^ exacerbated by subsequent immune checkpoint inhibition,^55,56^ which was not studied in our experimental setting.

Abatacept, a CTLA-4 fusion protein inhibits T-cell costimulation and is a promising therapeutic option for ICI-induced myocarditis.^16,17,57^ Moreover, in pre-clinical studies, abatacept was shown to alleviate pressure-overload-induced and ageing-related heart failure in mice.^18,25^ In our pre-clinical study, coadministration of abatacept prevented the decline in cardiac systolic function seen with anti-PD-1 treatment and attenuated the upregulation of pro-inflammatory markers. Moreover, abatacept increased the expression of PD-1 in the thymus in mice without prior ischaemic injury as a potential negative regulatory effect, while in mice with prior ischaemic injury and anti-PD-1 treatment, PD-1^+^ T cells were already increased in the thymus, and the pathway was not impacted by abatacept.

Limitations of our study include the use of a pre-clinical model without the concomitant presence of a tumour, which may affect the systemic response of ICI therapy. Nevertheless, as shared antigens between the tumour and myocardium have been suggested as a potential mechanism for ICI myocarditis, here, we were able to study the cardiac effects of immune checkpoint inhibition independently of tumour presence. Moreover, our parallel clinical study had a retrospective design. To investigate the effect of previous cardiac ischaemic events independently of other risk factors, multivariate logistic regression and propensity score matching were performed. Nevertheless, unmeasured confounders may affect the association. As a further sensitivity analysis, ICI-treated patients were matched to non-ICI-treated patients, where the strength of association between previous ischaemic events and heart failure development was weaker, further suggesting the potential role of myocardial ischaemic injury in heart failure development after ICI therapy.

Translational perspective Immune checkpoint inhibitors (ICIs) are increasingly used for the treatment of many cancer types, but may cause various cardiac toxicities. In this study, we show that previous cardiac ischaemic injury is associated with new-onset heart failure after anti-programmed death protein 1 treatment in a pre-clinical model and a parallel retrospective clinical study. Based on our findings, cancer patients with pre-existing ischaemic heart disease may be at higher risk for ICI-induced cardiac dysfunction, and cardiac surveillance should be considered in patients starting ICI therapy. Abatacept is a potential therapeutic option to alleviate the cardiovascular toxicities of ICIs.

## 5. Conclusions

In this translational study, induction of cardiac inflammation and dysfunction by anti-PD1 therapy was potentiated by prior transient ischaemic cardiac injury in mice. This effect was attenuated by cotreatment with abatacept. In our parallel retrospective clinical study, a history of ischaemic cardiac events was associated with new-onset heart failure after ICI treatment in a matched cohort (*Graphical Abstract*). In summary, patients with pre-existing ischaemic heart disease may be at greater risk for developing ICI-induced cardiotoxicity, including new-onset heart failure, whereas abatacept may be a promising therapeutic strategy. Based on these, cardiac surveillance may be considered in patients starting ICI therapy with a prior history of ischaemic heart disease.

## Supplementary Material

cvag085_Supplementary_Data

## Data Availability

The data from the pre-clinical study are available from the corresponding author upon reasonable request.
