# Supplementary material for: Inhibition of the programmed death protein 1 immune checkpoint and the development of heart failure in the presence of prior cardiac ischaemia
Source: Cardiovasc Res. 2026 Apr 22;122(8):1055–69. doi: 10.1093/cvr/cvag085 (PMC13238740; doi:10.1093/cvr/cvag085)
Supplement: cvag085_Supplementary_Data [file cvag085_supplementary_data.zip › ICI_prior_ischemia_HF_Supplementary_REVISION_20251007.docx]

**SUPPLEMENTARY MATERIALS**

**SUPPLEMENTARY METHODS**

**Echocardiography**

Mice were anesthetized with isoflurane (5% for induction, 2% for maintenance) and placed on heating pads maintaining 37°C body temperature, with continuous monitoring via rectal probe. Echocardiographic analysis was done with Vevo 3100 high-resolution in vivo imaging system (Fujifilm VisualSonics, Toronto, Canada) using an ultrahigh frequency MX400 transducer (30 MHz, 55 frames per second), by an operator blinded to the study groups. On two-dimensional recordings of the short-axis at the mid-papillary muscle level, measured parameters included left ventricular internal diameter in systole and diastole (LVIDs and LVIDd, respectively), LV anterior wall thickness (AWT), posterior wall thickness (PWT). End-diastolic and end-systolic LV areas were measured from short- and long-axis two-dimensional B-mode recordings. Diastolic parameters were measured in the apical four-chamber view. Pulse-wave Doppler was used to determine early mitral inflow velocity (E), isovolumetric relaxation and contraction time (IVRT and IVCT, respectively), and aortic ejection time (AET). Tissue Doppler imaging was used to quantify mitral annular early diastolic velocity (e’).

Fractional shortening (FS) was calculated as [(LVEDD − LVESD)/LVEDD] × 100. End-diastolic (LVEDV) and end-systolic (LVESV) LV volumes were calculated from the rotational volumes of the left ventricular trace at the diastole and systole around the long axis line of the spline. Stroke volume (SV) was calculated as LVEDV − LVESV. Ejection fraction (EF) was determined as (SV/LVEDV) × 100. Cardiac output (CO) was calculated as SVxHR/1000). Cardiac index was calculated as CO/body surface area (BSA, defined as 9.8 × body weight^2/3^). Volumes as dimensions were indexed to BSA in case of significant differences in body weight^1^. LV mass was calculated according to a cubic formula, suggested by Devereux et al. ^2^ and modified for rodents ^3^ (LV mass = 1.04 [(LVIDd+LVAWd+LVPWd)^3^- LVIDd^3^] x 0.8 + 0.6). Left ventricular remodeling index (LVRi) was calculated as LV mass / LVIDd. Myocardial performance index (MPI or Tei index) was calculated as (IVRT + IVCT) / AET.

Echocardiographic recordings were evaluated via VevoLAB software (Fujifilm VisualSonics, Toronto, Canada) by an evaluator blinded to the study groups. Recordings with a heart rate lower than 450 min^-1^ were excluded from analysis^1^. Global longitudinal strain (GLS) was assessed by speckle-tracking. For this, two to three consecutive cardiac cycles in B-Mode were selected. Using the Vevo Strain Software, semi-automated tracing of the endocardial and epicardial borders in the parasternal long-axis was performed to calculate GLS. Papillary muscles were excluded from GLS analysis. Mice with high respiratory rate which prevented tracing of consecutive cardiac cycles were excluded from GLS analysis.

**Histology**

Heart tissues were fixed in neutral buffered formalin for 24 hours, then dehydrated, embedded in paraffin. 5 μm thick sections were used for histological analyses and immunohistochemistry. All the stainings were visualized and images were captured with Leica LMD6 microscope (Wetzlar, Germany).

*Hematoxylin and eosin staining.* After initial de-paraffinization and hydration, heart sections were stained with hematoxylin and counterstained with eosin to evaluate morphologic and pathologic alterations.

*Sirius red staining.* After initial preparations (see above), heart sections were pre-treated with 0.2% aqueous phosphomolybdic acid, followed by staining with 0.0125% picrosirius red for 1 hour, and then washed with 0.01N HCl. An investigator blinded to study groups analyzed and quantified the extent of cardiac fibrosis using predefined threshold values using ImageJ Software.

*Immunohistochemistry.* Prepared heart sections underwent antigen retrieval (Antigen Unmasking Solution, Citrate-based, pH = 6, Vector Laboratories, Newark, CA, USA, H-3300 or Antigen Unmasking Solution, Tris-Based, pH = 9, Vector Laboratories, Newark, CA, USA, H-3301) for 30 min. Endogenous peroxidase activity was blocked by 3% H_2_O_2_ in PBS solution, and then the sections were blocked with 2.5% goat serum in PBS and 2.5% milk powder. Primary antibodies were diluted in goat serum (2.5%) and incubated overnight at 4°C. The following primary antibodies were used: anti-PD-L1 (1:400, rabbit IgG, NBP1-76769, Novus Biologicals), anti-Iba1 (1:2000, PTK1381, Fujifilm), anti-CD3ε (1:300, rabbit IgG, #99940, Cell Signaling Technology), anti-CD4 (1:100, rabbit IgG, #25229, Cell Signaling Technology), anti-CD8 (1:400, rabbit IgG, #98941, Cell Signaling Technology), anti-Foxp3 (1:400, rabbit IgG, #12653, Cell Signaling Technology). After washing with gentle shaking three times with PBS, the sections were incubated with anti-rabbit IgG secondary antibodies (8114S, Cell Signaling Technology, Leiden, The Netherlands) conjugated with horseradish peroxidase. Secondary antibodies were washed with gentle shaking three times with PBS, and signals were developed with diaminobenzidine (SK-4103, ImmPACT DAB EqV Peroxidase [HRP] Substrate, Vector Laboratories, Burlingame, CA, USA).

**RNA isolation and quantitative reverse transcription-polymerase chain reaction (qRT-PCR)**

Total RNA was isolated from mouse heart samples with a chloroform/isopropanol precipitation method. Briefly, Qiazol® (Qiagen, The Netherlands) was added to each sample and homogenized with TissueLyser (Qiagen, The Netherlands). Homogenates were centrifuged, and from the clean upper phase, DNA and protein were precipitated with chloroform. Total RNA was extracted with isopropanol, and pellets were washed four times with 75% ethanol (vWR, PA, US). Finally, total RNA was resuspended in nuclease-free water, and RNA concentration was determined by spectrophotometry (Implen Nanophotometer® N60, München, Germany). cDNA was synthesized from 1 µg total RNA by Sensifast cDNA synthesis kit (Bioline, UK) according to the manufacturer’s protocol. cDNA was further diluted 20x with RNAse free water. qRT-PCR reactions were performed on a LightCycler® 480 II instrument (Roche, Germany) by using SensiFAST SYBR Green master mix (Bioline, UK). Sequences of primers are shown in Supplementary Table 1. Ribosomal protein L13a (Rpl13a) was used as housekeeping gene. Results were calculated with 2‐ΔΔCp evaluation method.

**Supplementary Table 1.** Sequences of primers used for qRT-PCR.

| **Gene name** | **Accession number** | **Forward primer** | **Reverse primer** | **Product size (bp)** |
| --- | --- | --- | --- | --- |
| *Ccl5* | NM_013653.3 | GTGCCCACGTCAAGGAGTAT | TCGAGTGACAAACACGACTG | 71 |
| *Cd274 (PD-L1)* | NM_021893.3 | CAGCAACTTCAGGGGGAGAG | TTTTGCGGTATGGGGCATTG | 176 |
| *Cd3e* | NM_007648.5 | GCGTCTGGTGCCTTCTTCAG | CAGGATGCCCCAGAAAGTGT | 108 |
| *Cd68* | NM_001291058.1 | TGCGGCTCCCTGTGTGT | TCTTCCTCTGTTCCTTGGGCTAT | 61 |
| *Foxp3* | NM_001199347.1 | AGAGGTATTGAGGGTGGGTGT | TCTAAGCTTTCTTCTGTCTGGAGTG | 80 |
| *Gzmb* | NM_013542.3 | CTGCTCACTGTGAAGGAAGTATAA | AGCTCTAGTCCTCTTGGCCT | 185 |
| *Icam1* | NM_010493.3 | AACTTTTCAGCTCCGGTCCTG | TCAGTGTGAATTGGACCTGCG | 154 |
| *Ifnγ* | NM_008337.4 | CGGCACAGTCATTGAAAGCC | TGCATCCTTTTTCGCCTTGC | 268 |
| *Il17a* | NM_010552.3 | ACTACCTCAACCGTTCCACG | TTCCCTCCGCATTGACACAG | 120 |
| *Il1ß* | NM_008361.4 | GCACTACAGGCTCCGAGATGAAC | TTGTCGTTGCTTGGTTCTCCTTGT | 147 |
| *Il23* | NM_031252.2 | AATGCTATGGCTGTTGCCCT | AGACCTTGGCGGATCCTTTG | 270 |
| *Il6* | NM_031168.2 | GACAAAGCCAGAGTCCTTCAGAGAG | CTAGGTTTGCCGAGTAGATCTC | 228 |
| *Itgal* | NM_001253872.1 | GCCACAGTTTCAAGGTCGC | AGGTCTCAGGATAGGCTGCAT | 286 |
| *Klrb1* | NM_001099918.2 | AGGACTGAACCAACAGGGAGAT | GTCAACCAGACCGTCAGCC | 120 |
| *Nkg7* | NM_024253.4 | GCCTGGTGTCTGTGAGCTTC | TAGCCACTAATATGGAGAGAGCTGC | 120 |
| *Pdcd1 (PD-1)* | NM_008798.3 | CAAGGACGACACTCTGAAGGAG | TCTTCTCTCGTCCCTGGAAGT | 89 |
| *Tnfα* | NM_013693.3 | TACTGAACTTCGGGGTGATTGGTCC | CAGCCTTGTCCCTTGAAGAGAACC | 295 |

**Enzyme-linked immunoassay (ELISA)**

The concentration of cardiac Troponin-I (cTNI) in the mouse serum samples was detected by Ultrasensitive Mouse Cardiac Troponin-I ELISA kit (CTNI-1-US, Life Diagnostics, West Chester, PA, USA) according to the manufacturer’s instructions. Briefly, microplates were precoated with anti-cTNI antibodies. Standards were prepared from reconstituted cTNI stock, then samples and standards were dispensed into the wells and incubated 2 hours at room temperature on a plate shaker. After washing, HRP-Conjugate was added to each well and incubated for a further 1 hour at room temperature. After addition of Substrate Solution (TMB) and Stop Solution, optical density was determined immediately, using a microplate reader set to 450 nm. cTNI concentrations (ng/ml) of the serum samples were calculated from the optical density of the standard curve by a curve fitting software.

**Western blot**

To investigate whether PD-1 expression was altered at the protein level in mouse thymus samples, Western blot was performed. Frozen tissue samples were homogenized with a TissueLyser (Hilden, Germany) in 1× radio immunoprecipitation assay buffer (RIPA; Cell Signaling Technology, Danvers, MA, USA) supplemented with 1× HALT Protease and Phosphatase Inhibitor cocktail (Thermo Scientific, Waltham, MA, USA). Protein concentration of the samples was determined by bicinchoninic acid assay kit (Thermo Scientific, Waltham, MA, USA). Equal amounts of protein (25 µg) from each sample were mixed with 1/4 volume of Laemmli buffer containing β-mercaptoethanol (Thermo Scientific, Waltham, MA, USA) and were loaded on 4–20% Tris-glycine sodium dodecyl sulfate-polyacrylamide gels (Bio-Rad, Hercules, CA, USA). After separation by electrophoresis, proteins were transferred onto polyvinylidene difluoride membrane (Bio-Rad, Hercules, CA, USA) with Trans-Blot^®^ Turbo^TM^ Transfer System (Bio-Rad, Hercules, CA, USA). Membranes were blocked in 5% bovine serum albumin (Bio-Rad, Hercules, CA, USA) in Tris-buffered saline containing 0.05% Tween-20 (0.05% TBS-T; Sigma, St. Louis, MO, USA) for 2 h at room temperature. Afterward, membranes were incubated with anti-PD-1 primary antibody overnight at 4 °C (1:1000 dilution). After three washes in TBS-T, membranes were incubated with HRP-conjugated anti-rabbit secondary antibodies (Cell Signaling, Danvers, MA, USA) for 2 h and washed in TBS-T. Signals were visualized after incubation with an enhanced chemiluminescence kit (Bio-Rad, Hercules, CA, USA) by Chemidoc XRS+ (Bio-Rad, Hercules, CA, USA). Image analysis was performed using Image Lab™ 6.0 software (Bio-Rad, Hercules, CA, USA). Measurement of GAPDH content was used as loading control. Briefly, membranes were incubated with Restore Stripping Buffer for 15 min at room temperature (Thermo Scientific, Waltham, MA, USA), followed by incubation with anti-GAPDH primary antibody (1:5000 dilution, overnight at 4 °C), HRP-conjugated anti-rabbit secondary antibody (1:2000 dilution, 2 h at room temperature), and signal detection, as described previously.

**Flow cytometry**

The thymus and the spleen were manually dissected and homogenized through a 40 µm cell strainer (pluriStrainer 40μm, Nylon-Mesh. 43-57040-51). The samples were centrifuged for 5 minutes at 400x*g* and resuspended in 0.5-1 ml PBS. Samples were stained with the primary antibodies or isotype controls for 15 minutes at room temperature (Supplementary Table 2). The spleen samples were incubated with 1 mL red blood cell lysis buffer for 10 minutes (eBioscience™ 1X RBC Lysis Buffer, 00-4333-57). After washing steps all samples were fixed in 2% PFA. A CytoFlex S flow cytometer (Beckman Coulter, Indianapolis, IN, USA) was used for data acquisition and data were analysed using the CytExpert software (Beckman Coulter, Indianapolis, IN, USA) by an evaluator blinded to study groups.

| **Supplementary Table 2.** Antibodies and isotype controls for flow cytometry experiments. | | |
| --- | --- | --- |
| **Name** | **RRID** | **CAT NO** |
| **Antibodies** |  |  |
| CD3 (17A2) Rat mAb (PE) | AB_2798955 | CST 28306 |
| CD4 (RM4-5) Rat mAb (violetFluor 450) |  | CST 92599 |
| CD8-Alpha(2.43) Rat mAb (PE-Cy7) | AB_2800109 | CST 87922 |
| CD69 (H1.2F3) Hamster mAb (FITC) |  | CST 39443 |
| APC Rat Anti-Mouse CD25 |  | CST 32325 |
| APC Hamster Anti-Mouse CD279 (PD-1) | AB_2737712 | BD 562671 |
| Alexa Fluor 488 Rat Anti-Mouse CD274 | AB_2869917 | BD 566864 |
| APC-R700 Rat Anti-Mouse CD62L | AB_2737397 | BD 565159 |
| CD4-Vio Blue, mouse Clone REA604 |  | 130-120-674 |
| CD25-APC, mouse Clone REA568 |  |  |
| Anti-FoxP3-PE, mouse Clone REA788 |  |  |
|  |  |  |
| **isotype controls** |  |  |
| APC Rat IgG1, λ Isotype Control | AB_10050452 | BD 550884 |
| FITC Armenian Hamster IgG Isotype Control |  | BZ-400906 |
| APC Hamster IgG2, κ Isotype Control | AB_10896490 | BD 562169 |
| Alexa Fluor 488 Rat IgG2a, κ Isotype Control | AB_396787 | BD 557676 |
| APC-R700 Rat IgG2a, κ Isotype Control | AB_2869633 | BD 564982 |
| REA Control Antibody (1) , human IgG1, PE, REAfinity™ Clone REA293 | AB_2857433 | 130-118-347 |

| **SUPPLEMENTARY RESULTS**  **Supplementary Table 3.** Baseline data for non-ICI treated patients. | | | | |
| --- | --- | --- | --- | --- |
|  |  | **No HF** | **New HF** |  |
|  | **n** | **n = 648** | **n = 103** | **p-value** |
| **Demographics** |  |  |  |  |
| **Gender**– no. (%) | 751 |  |  | 0.8 |
| Female |  | 305 (47%) | 47 (46%) |  |
| Male |  | 343 (53%) | 56 (54%) |  |
| **Age**– yr, median. (IQR) | 751 | 64 (54, 72) | 70 (60, 77) | **<0.001** |
| **Race** – no. (%) | 751 |  |  | 0.3 |
| Asian |  | 18 (2.8%) | 3 (2.9%) |  |
| Black or African American |  | 10 (1.5) | 2 (1.9) |  |
| Other |  | 31 (4.8%) | 5 (4.9%) |  |
| White |  | 589 (91%) | 93 (90%) |  |
| Body mass index | 751 | 26.1 (23.1, 30.1) | 25.7 (23.4, 29.7) | >0.9 |
| **Cancer type** – no. (%) | 751 |  |  |  |
| Breast |  | 23 (3.5%) | 2 (1.9%) |  |
| Gastroinestinal |  | 71 (11%) | 10 (9.7%) |  |
| Genitourinary |  | 34 (5.2%) | 7 (6.8%) |  |
| Gynecologic |  | 30 (4.6%) | 3 (2.9%) |  |
| Head and neck |  | 70 (11%) | 12 (12%) |  |
| Hematologic |  | 15 (2.3%) | 6 (5.8%) |  |
| Melanoma |  | 162 (25%) | 26 (25%) |  |
| Neurological |  | 8 (1.2%) | 0 (0%) |  |
| Thoracic |  | 235 (36%) | 37 (36%) |  |
| **Cardiovascular risk factors and comorbidities** – no. (%) | | | | |
| Arrhythmia | 751 | 196 (30%) | 51 (50%) | **<0.001** |
| Hypertension | 751 | 292 (45%) | 69 (67%) | **<0.001** |
| Diabetes mellitus | 751 | 24 (3.7%) | 6 (5.8%) | 0.3 |
| Renal disease | 751 | 64 (9.9%) | 17 (17%) | **0.044** |
| Liver disease | 751 | 219 (34%) | 32 (31%) | 0.6 |
| Hyperlipidemia | 751 | 234 (36%) | 51 (50%) | **0.009** |
| Smoking | 751 | 103 (16%) | 21 (20%) | 0.3 |
| **Anticancer therapy** – no. (%) | | | | |
| Antracycline | 751 | 40 (6.2%) | 4 (3.9%) | 0.4 |
| 5-FU | 751 | 34 (5.2%) | 5 (4.9%) | 0.9 |
| Platin | 751 | 203 (31%) | 36 (35%) | 0.5 |
| Tyrosine kinase inhibitor | 751 | 29 (4.5%) | 3 (2.9%) | 0.6 |
| **Cardiovascular drug therapy** – no. (%) | | | | |
| Angiotensin-converting-enzyme inhibitors | 751 | 113 (17%) | 18 (17%) | >0.9 |
| Angiotensinr eceptor blocker | 751 | 41 (6.3%) | 10 (9.7%) | 0.2 |
| Beta blocker | 751 | 170 (26%) | 45 (44%) | **<0.001** |
| Calcium channel blocker | 751 | 69 (11%) | 26 (25%) | **<0.001** |
| Diuretics | 751 | 141 (22%) | 38 (37%) | **<0.001** |
| Statin | 751 | 151 (23%) | 28 (27%) | 0.4 |
| Antiarrhytmic | 751 | 46 (7.1%) | 13 (13%) | **0.053** |
| Antiplatelet | 751 | 15 (2.3%) | 3 (2.9%) | 0.7 |
| Aspirin | 751 | 96 (15%) | 32 (31%) | **<0.001** |
| Low molecular weight heparin | 751 | 64 (9.9%) | 10 (9.7%) | >0.9 |
| Novel oral anticoagulant | 0 | 0 (NA%) | 0 (NA%) |  |
| Warfarin | 751 | 55 (8.5%) | 11 (11%) | 0.5 |
| **Cardiovascular events** – no. (%) | | | | |
| Prior coronary artery bypass grafting | 751 | 13 (2.0%) | 6 (5.8%) | **0.035** |
| Prior percutaneous coronary intervention | 751 | 16 (2.5%) | 4 (3.9%) | 0.3 |
| Prior revascularization | 751 | 27 (4.2%) | 8 (7.8%) | 0.13 |
| Prior myocardial infarction | 751 | 19 (2.9%) | 7 (6.8%) | 0.073 |
| Prior stroke | 751 | 16 (2.5%) | 3 (2.9%) | 0.7 |
| Prior cardiac ischemic event (myocardial infarctuin, PCI or CABG) | 751 | 34 (5.2%) | 10 (9.7%) | 0.073 |

**FIGURES**

**
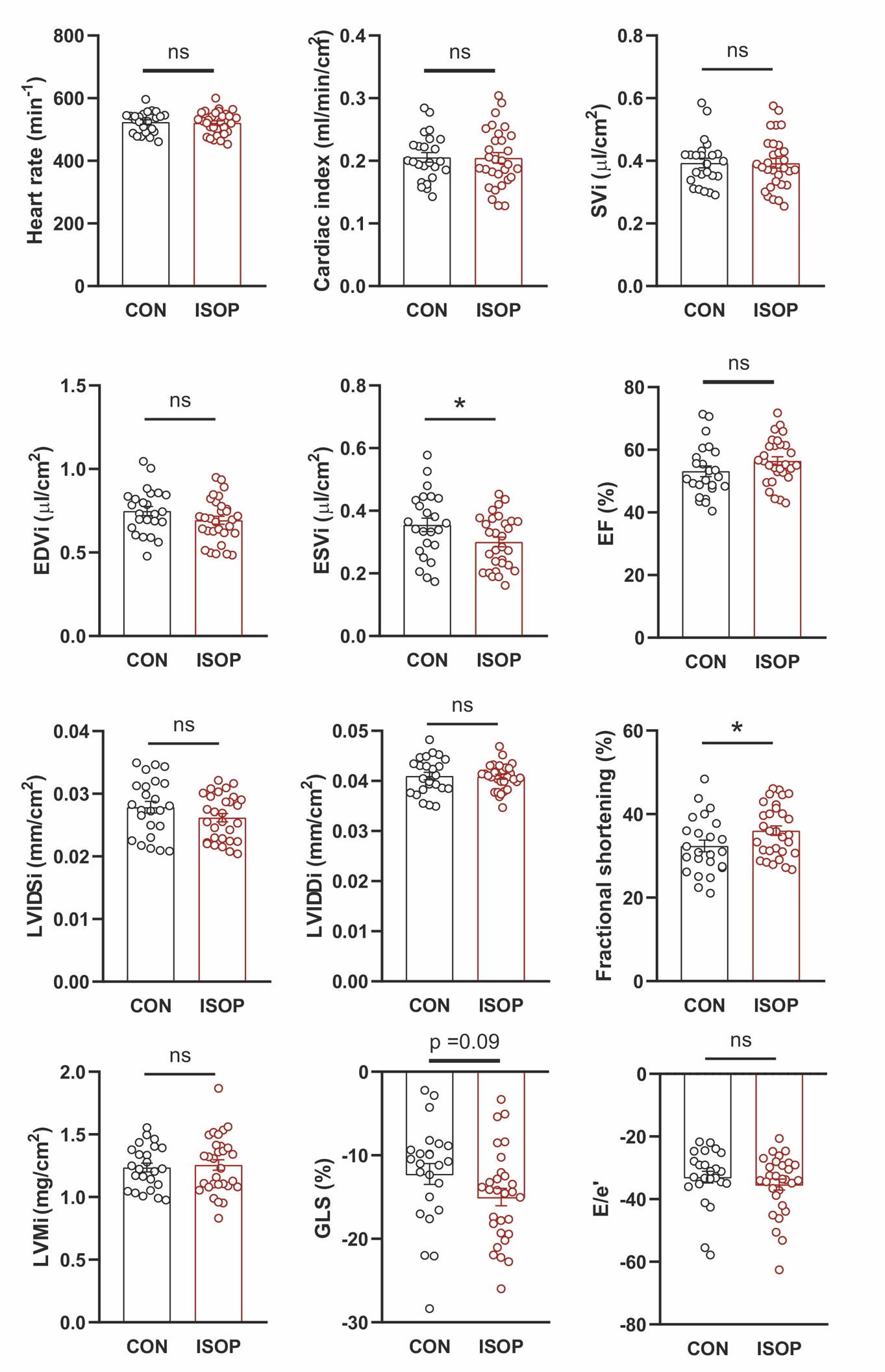
**

**Supplementary Figure 1. Echocardiographic measurements after 16 weeks of follow-up.**

* p < 0.05 vs. CON group, Student’s t-test. Abbreviations: SVi: stroke volume index, EDVi: end-diastolic volume indexed to BSA, ESVi: end-systolic volume indexed to BSA, EF: ejection fraction, LVIDSi: left ventricular internal diameter at systole indexed to BSA, LVIDDi: left ventricular internal diameter at diastole indexed to BSA, LVMi: left ventricular mass indexed to BSA, GLS: global longitudinal strain, E/e’: ﻿early mitral inflow velocity (E) and mitral annular early diastolic velocity, IVRT: isovolumetrix relaxtion time


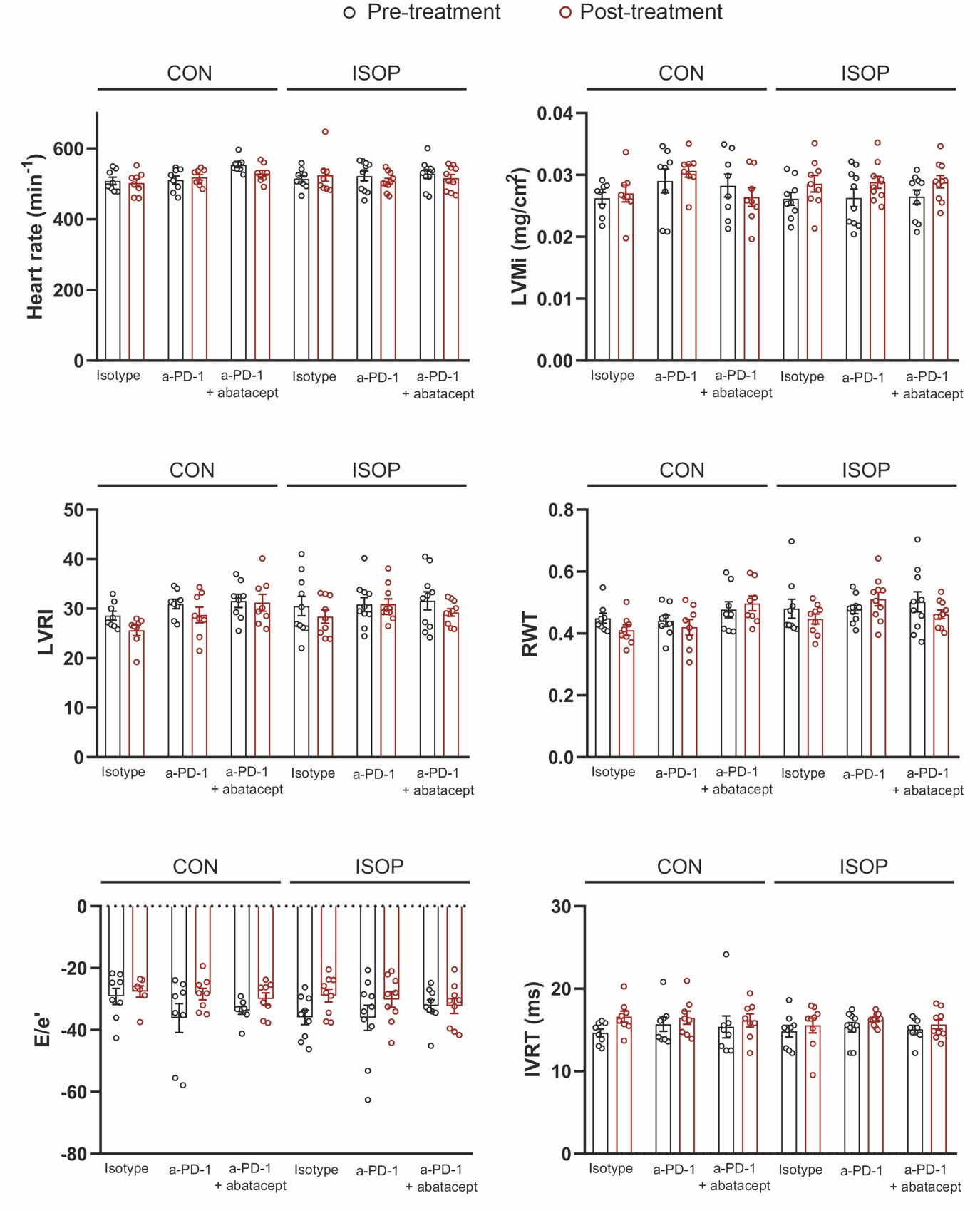
**Supplementary Figure 2. Echocardiographic measurements before and after treatment with either isotype control, anti-PD-1 or anti-PD-1 + abatacept.** No significant differences was observed with repeated measures ANOVA between pre-and post-treatment groups.
Abbreviations: LVMi: left ventricular mass indexed to BSA, LVRi: left ventricular remodelling index, RWT: relative wall thickness, E/e’: ﻿early mitral inflow velocity (E) and mitral annular early diastolic velocity, IVRT: isovolumetrix relaxtion time


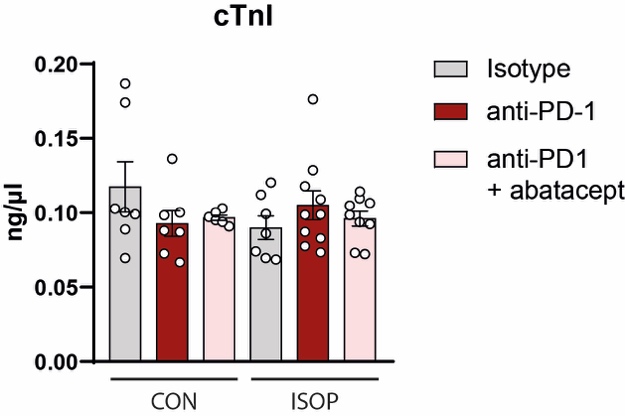


**Supplementary Figure 3. Cardiac troponin I measurement via ELISA.** No significant differences was observed with Kruskal-Wallis test. n = 6-8/group for CON groups, n = 6-9/group for ISOP groups.

**
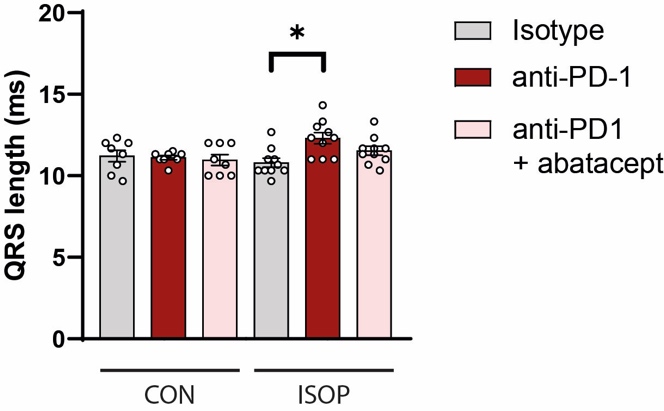
**

**Supplementary Figure 4. Length of QRS on ECG measurements.** * p < 0.05, Kruskal-Wallis test followed by Dunn’s post hoc test, n=8/group for CON groups, n=10 for ISOP groups.

**Supplementary Figure 5. Flow cytometric quantification of PD1+ Treg cells in the thymus. (A)** Gating strategy. **(B)** Anti-PD-1 treatment decreased the % of PD1+ Treg cells in the thymus. * p < 0.05, unpaired t-test with Welch’s correction, n = 4 / group.

**REFERENCES**

1. Zacchigna, S. *et al.* Towards standardization of echocardiography for the evaluation of left ventricular function in adult rodents: A position paper of the ESC Working Group on Myocardial Function. *Cardiovasc Res* 117, 43–59 (2021).

2. Richard B. Devereux, Daniel R. Alonso, Elizabeth M. Lutas, Geoffrey J. Gottlieb, Emilio Campo, Irene Sachs, Nathaniel Reichek. Echocardiographic assessment of left ventricular hypertrophy: Comparison to necropsy findings. *Am J Cardiol* 57, 450–458 (1986).

3. Zacchigna, S. *et al.* Towards standardization of echocardiography for the evaluation of left ventricular function in adult rodents: A position paper of the ESC Working Group on Myocardial Function. *Cardiovasc Res* 117, 43–59 (2021).
