# Supplementary material for: Inhibition of the programmed death protein 1 immune checkpoint and the development of heart failure in the presence of prior cardiac ischaemia
Source: Cardiovasc Res. 2026 Apr 22;122(8):1055–69. doi: 10.1093/cvr/cvag085 (PMC13238740; doi:10.1093/cvr/cvag085)
Supplement: cvag085_Supplementary_Data [file cvag085_supplementary_data.zip › Supplementary_Figure_Unedited_Western_blot_gels.docx]

**Unedited Western blot gels**

**
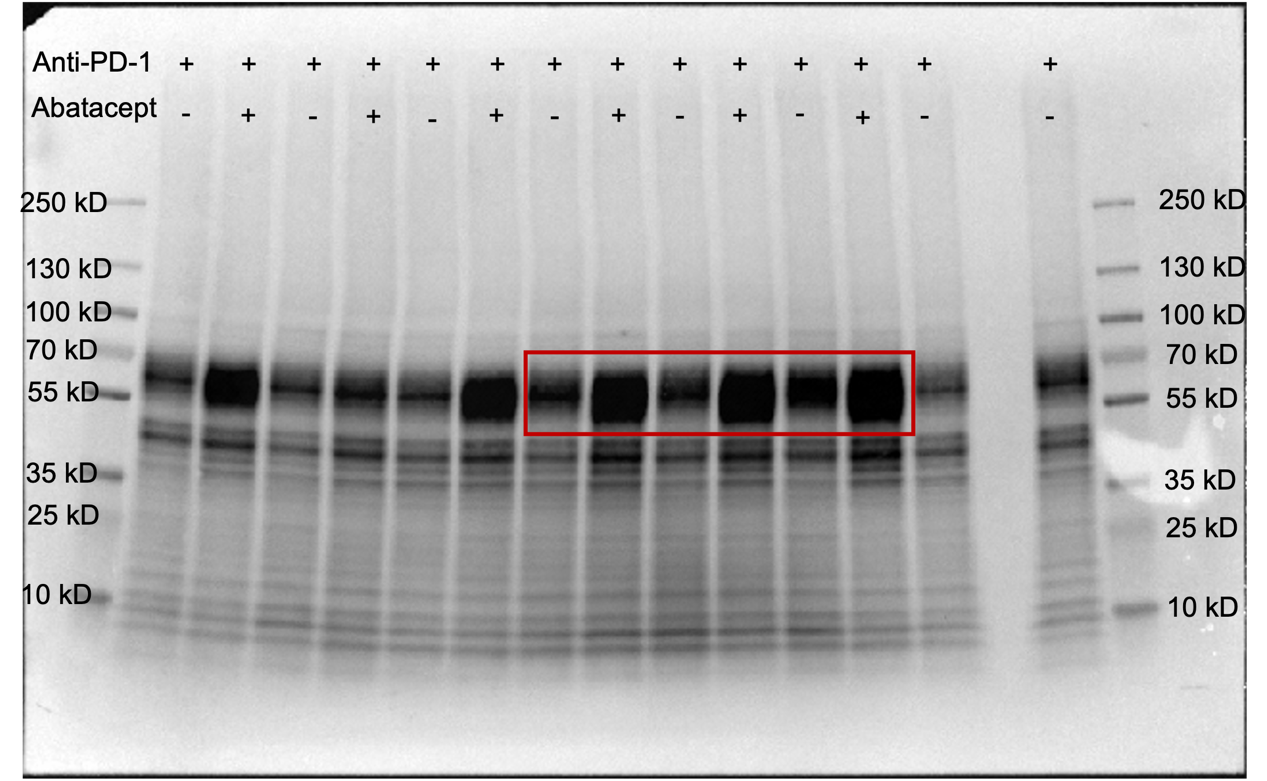
PD-1**

**
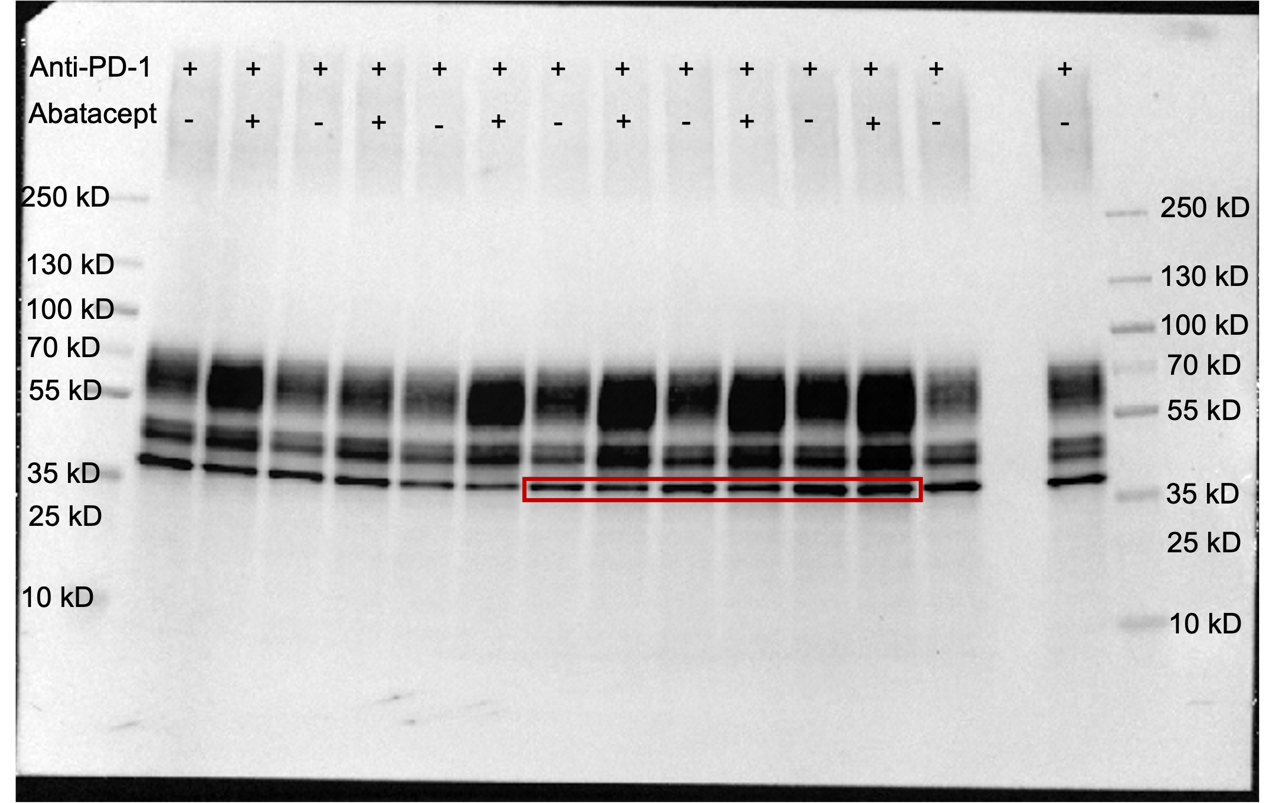
GAPDH**

Red highlights show the part corresponding with cropped images in Figure 4/D.
